# Comprehensive genomic analysis of a novel *Bacillus cereus* decomposing toluene potentially applicable in bioremediation

**DOI:** 10.1128/spectrum.02892-24

**Published:** 2025-12-01

**Authors:** Maryam Safari, Samira Ghahroodian, Mohammad vahid Abyarazimi, Samira Rahmaniyan, Fatemeh Heydaryan, Marzieh Rezaei, Bagher Yakhchali

**Affiliations:** 1 Department of Biology, Faculty of Science, Nour Danesh Institute of Higher Education, Meymeh, Iran; 2 Institute of Industrial and Environmental Biotechnology, National Institute of Genetic Engineering and Biotechnology (NIGEB) 48482, Tehran, Iran; Institute of Microbiology, Chinese Academy of Sciences, Beijing, China

**Keywords:** *Bacillus cereus*, bioremediation, Taguchi method, meta-cleavage pathway, toluene, pan-genome analysis

## Abstract

Environmental pollution treatment is a worldwide challenge. Biological treatments are preferred to remedy the pollutants owing to higher efficiency and biocompatibility. Here, 18 indigenous bacterial strains with the usability of toluene as the sole carbon and energy source were screened, resulting in the selection of one isolate that was most capable of removing toluene. This isolate was characterized as a rod-shaped, gram-positive, spore-forming, halotolerant, catalase-positive, motile bacterium. The optimal temperature (40°C), pH (8), and NaCl concentration (50 g/L) for toluene removal were determined. Then, the experiment with four variables (petrochemical effluent dilution, carbon source, minerals, and amount of municipal wastewater) was designed using a Taguchi L16 orthogonal array. Ultimately, a 38.15% chemical oxygen demand decrease, as the best removal efficiency, was achieved. Gas chromatography–mass spectrometry analysis of bacterial metabolites assisted in recognizing the toluene degradation products, such as acetate and 2-propanone, indicating a meta-pathway of toluene decomposition. Next-generation sequencing strategy followed by core/pan-genome analysis indicated that the genome of the strain encompasses an assembly size of 5,154,622 bp with a content of 35.34% comprising 3,437 core and 2,258 accessory genes. Phylogenetic and multi-locus sequence typing studies confirmed that the strain belongs to the genus *Bacillus* and the species *cereus*. All the annotated and anticipated coding sequences were allocated to Kyoto Encyclopedia of Genes and Genomes routes. Accordingly, genes contributing to the toluene degradation pathway, including genes coding catechol 2, 3-dioxygenase, 4-oxalocrotonate isomerase, 2-hydroxymuconate-semialdehyde hydrolase, 2-keto-4-pentenoate hydratase, acetaldehyde dehydrogenase, alcohol dehydrogenase, flavocytochrome P450, anthranilate 3-monooxygenase (flavin adenine dinucleotide [FAD]), and flavin-dependent oxidoreductase, were ascertained. Herein, a general view of the GYRND102 strain is presented, which led to providing further insight into the decomposition mechanisms of aromatic pollutants by the strain for its effective application at the industrial level. Therefore, using this strain for pollutant removal will be cost-effective due to the simplicity of the method and the fact that it requires no special facilities or equipment.

**IMPORTANCE:**

The purpose of the present study was to develop a feasible process for the treatment of heavily contaminated Mahshahr petrochemical effluent. Since regular treatment of this effluent was inefficient, isolation of an indigenous bacterium with the ability to decompose toluene (the main pollutant of the effluent) and use it as a biological material to treat wastewater containing aromatic compounds on an industrial scale was very important.

## INTRODUCTION

Aromatic compounds and their derivatives have been known as the top 10 most prevalent pollutants in water and soil because of their widespread use in many industries (1). Therefore, environmental contamination poses a major menace to the ecosystem, which widely affects all organisms and human health (2). Bioremediation of environmental pollutants and polluted sites can be accomplished by some microorganisms such as bacteria, which need to form a close physical association with the contaminants (3). Aromatic compounds such as benzoate, toluene, and phenol are attractants for many bacteria (3, 4). Due to low costs and low-tech features, the biological treatment of polluted sites is highly valuable (1). Hence, bioremediation can be considered as a kind of medication for the environment in which aromatic-degrading microorganisms isolated from the same or similar contaminated environments are used (4). Accordingly, indigenous microorganisms are preferred for contaminant remediation because they can endure unfavorable conditions, are adapted to contaminated environments (2), use these compounds as a carbon source, and have a greater capacity to degrade pollutants into non-toxic and simpler substrates (5).

Toluene (methylbenzene) is a carcinogenic volatile organic compound and a major air pollutant that poses a risk to human health and the environment (6, 7). Owing to its high toxicity and degradation resistance, the U.S. Environmental Protection Agency classifies toluene as a priority pollutant (7). Accordingly, toluene has to be eliminated from polluted soil and water resources as well as industrial sites. As previously studied, bacteria play a substantial role in the degradation and mineralization of contaminants, including toluene, due to their fast growth rate, simple growth requirements, and adaptability to harsh environmental conditions (8, 9). Some examples of toluene-degrading bacteria include *Pseudomonas putida* (10), *Bacillus pumilus*, and *Bacillus cereus* (11).

Multiple catabolic enzymes transform aromatic compounds to Krebs cycle intermediates. In bacteria, two major biochemical cleavage pathways, the meta- and ortho-pathways, are utilized to cleave the catechol oxygenated ring and convert the subsequent substrates to Krebs cycle intermediates (12). Although catabolic pathways for breakdown of aromatic compounds by various bacteria may be varied, the final products are applied as sources of carbon and energy in the metabolic routes. Specifically, degradation of aromatic compounds and their conversion to catechol are carried out in reactions that are catalyzed by dioxygenase and monooxygenase enzymes (5). The catechol is the common intermediate of all pathways, which is metabolized by catechol 2,3-dioxygenase (meta-cleavage pathway) or by catechol 1,2-dioxygenase (ortho-cleavage pathway), leading to the production of simple compounds such as pyruvate (5, 13).

Moreover, the identification of genetic characteristics of bacteria (such as structural and regulatory genes of the metabolic pathways involved in environmental processes, as well as genetic mechanisms that contribute to the adaptive response of bacteria to environmental stimuli) isolated from the environment helps us better comprehend and predict their features and abilities in detail (14). Whole-genome sequencing using next-generation sequencing technologies, along with developing novel assembling methods and annotation analysis of the genomic data, paves the way for researchers to achieve this goal (15, 16).

Environmental contamination, mainly pollution with petroleum derivatives, is one of the main challenges in the Persian Gulf basin and oil-rich areas with high salt levels (17–20). In the last decade, many weakly, moderate, and extremely halophilic/halotolerant (2%–5%, 5%–20%, and 2%–30% NaCl, respectively) (21) bacterial strains have been isolated from different environments with the ability to decompose and use xenobiotic compounds such as crude oil, toluene, benzene, and chlorobenzene as the only sources of carbon and energy. These bacteria include *Amycolicicoccus subflavus*, *Marinobacter sedimentalis*, *Marinobacter falvimaris*, *Alcanivorax* sp., *Rhodobacter sphaeroides*, *Pseudomonas putida*, *Halomonas* sp., and *Marinobacter* sp. (19, 20, 22–27). A few microorganisms have been able to break down benzene, toluene, ethylbenzene, and xylene (BTEX) in high-salinity environments. Moreover, BTEXs as volatile compounds are less accessible and resistant to biodecomposition (17). Therefore, more studies to identify other microorganisms, especially bacteria with the catabolic potential to break down the desired pollutants, are requisite. To our knowledge, studies on the decomposition of hydrocarbons, including polycyclic aromatic hydrocarbons under ultra-saline conditions, are still insufficient and require further research (27–29). In the present study, the biodecomposition potential of indigenous *Bacillus cereus* GYRND102 under hypersaline conditions (50 g/L NaCl) gives it an advantage for its employment in on-site bioremediation of hydrocarbon-polluted locations under high-salinity levels.

The strains attributed to the *Bacillus* and *Pseudomonas* genera are known to be the most dominant strains in the decomposition of various pollutants due to their considerable biochemical versatility, increasing the solubility of pollutants by expressing and releasing biosurfactants, facilitating their following adsorption and degradation and their application to remedy different contaminated environments as widely reported (30–33).

Due to sporulation, biofilm formation, and osmotic adaptation, *Bacillus cereus* outstrips *Rhodococcus* and *Pseudomonas* in decomposing toluene in the presence of high salt concentration. The practical bioremediation potential of *Bacillus cereus* in saline environments has been confirmed. Since many toluene-polluted sites, such as saline soils and industrial wastewaters, restrain the growth of most bacteria due to their high salinity, *Bacillus cereus* can make use of diverse carbon sources, including aromatic hydrocarbons such as toluene, under the aerobic conditions present in these environments. Some strains of *cereus* can break down toluene through the xyl (xylene monooxygenase) or tod (toluene dioxygenase) pathways due to the presence of monooxygenase and dioxygenase enzymes. Additionally, *B. cereus* often enhances its capability to adhere to hydrophobic toluene droplets via biofilm formation, ameliorating degradation efficiency. This attribute can be efficient and beneficial in bioreactors and oil-contaminated aquatic systems (20, 34–40). Therefore, the *Bacillus cereus* halophilic/halotolerant strain is superior in degrading toluene in saline environments, while other genera may perform better just under optimal and low-salt conditions. Accordingly, *Bacillus cereus* is known as a promising candidate for bioremediation in marine, highly saline, and industrial effluent systems. Hence, the present study provides a detailed characterization of a strain identified as *Bacillus cereus*, isolated from industrial sites in Isfahan province, and evaluates its potential application in treating hydrocarbon-polluted areas under saline conditions. Since regular treatment of the heavily contaminated effluent from Mahshahr petrochemical was inefficient, isolation of an indigenous bacterium with the ability to decompose toluene and use it as a biological material to treat wastewater containing aromatic compounds on an industrial scale is very important.

**Fig 1:**
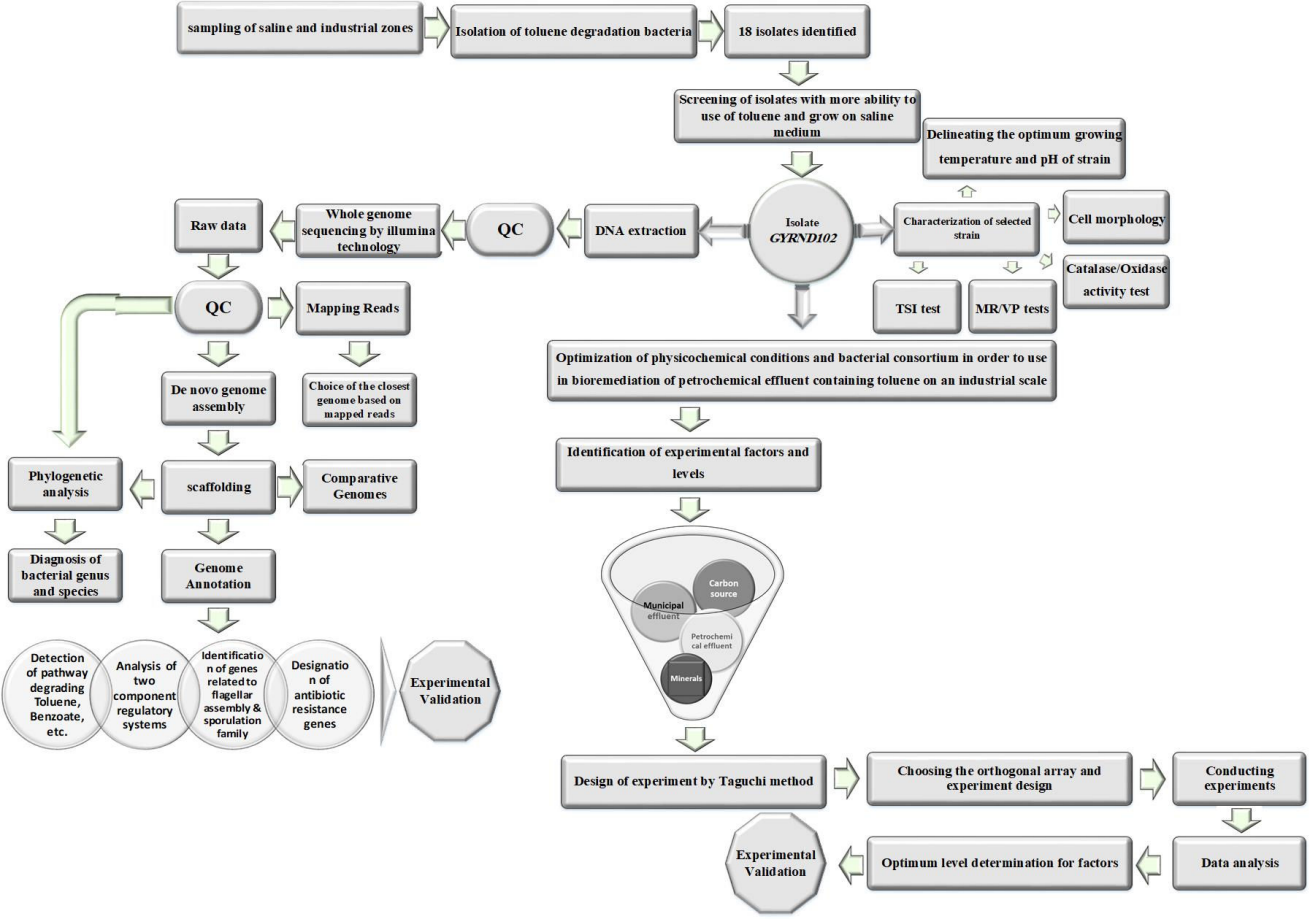
Workflow of the isolation and characterization of the *GYRND102* strain, as well as its genome sequencing and analysis. It consists of strain isolation, screening of the strain with ability to degrade toluene, cell morphology, petrochemical effluent characterization, and optimization of physicochemical conditions. As illustrated, experiments designed using the Taguchi method investigated the impact of four factors on bacterial ability to degrade pollutants. Moreover, the *GYRND102* genome was sequenced, and the resulting data were mapped against the reference genome after filtering and trimming and then used for phylogenetic analysis. Reads were *de novo* assembled to construct a draft genome assembly. Further analyses were performed on the resulting GYRND102 genome. MR, methyl red; TSI, triple sugar iron; VP, Voges-Proskauer.

Here, the toluene-degrading strain was genetically characterized, and its optimum conditions for the treatment of Mahshahr petrochemical effluents were studied (Fig. 1). Since this strain can grow rapidly in the vicinity of a pollutant with high salinity and does not require an inducer or a specific culture medium for the growth and decomposition of the aromatic pollutant, its cultivation and employment for industrial purposes will not need special facilities and much expense. Therefore, this strain usage can be very useful and cost-effective in industrial applications and in the bioremediation of high-salinity environments contaminated with aromatic pollutants.

## RESULTS

### Isolation and identification of bacterial strain

During the initial isolation, using random sampling from several industrial zones contaminated with toluene and enrichment procedures using toluene-containing basal medium (20% toluene [vol/vol] and NaCl [50 g/L]), 18 toluene-degrading/tolerant bacteria were isolated. Among those, a novel bacterium (bacterium A), isolated from a soil sample of the Isfahan petrochemical zone and showing a greater ability to degrade toluene, was selected for further studies. In total, the cultivation of bacteria in a minimal salt medium (MSM) containing different concentrations of toluene (1%–20% vol/vol) showed that all bacteria grew well in the presence of 10% toluene (vol/vol) by the turbidity measurement of the cultures. Based on data in Table S1, among all bacteria, bacterium A with 6% removal (out of 10%) was more able to remove toluene in a minimal salt medium containing 10% toluene (vol/vol). Accordingly, bacterium A was designated as a GYRND102 isolate and selected as the most potent isolate for further studies.

### Morphological, biochemical, and growth characteristics of the GYRND102 isolate

The isolate colonies were moderate in size, glossy, soft, creamy white, convex, and circular with entire margins (Fig. S1A), and bacterial cells were motile, gram positive, and rod shaped (Fig. S1B). According to the morphological and biochemical studies reported in Table 1, the GYRND102 isolate is very close to the *Bacillus* genus (41).

**TABLE 1:** Preliminary identification of isolate *A* (*GYRND102*)^*a*^^,^^*b*^

Tests	Characters
Gram staining	Positive
Morphology	Rod shaped
TSI	K/A
Citrate utilization	+
Hydrogen sulfide production	+
Indole production	+
Gas production	−
Nitrate reduction	−
MR	−
VP	−
Motility	+
Starch hydrolysis	−
Gelatin hydrolysis	+
Oxidase	−
Catalase	+

^*a*^ MR, methyl red; TSI, triple sugar iron; VP, Voges-Proskauer.

^*b*^ “+” indicates positive and “−” indicates negative.

**Fig 2:**
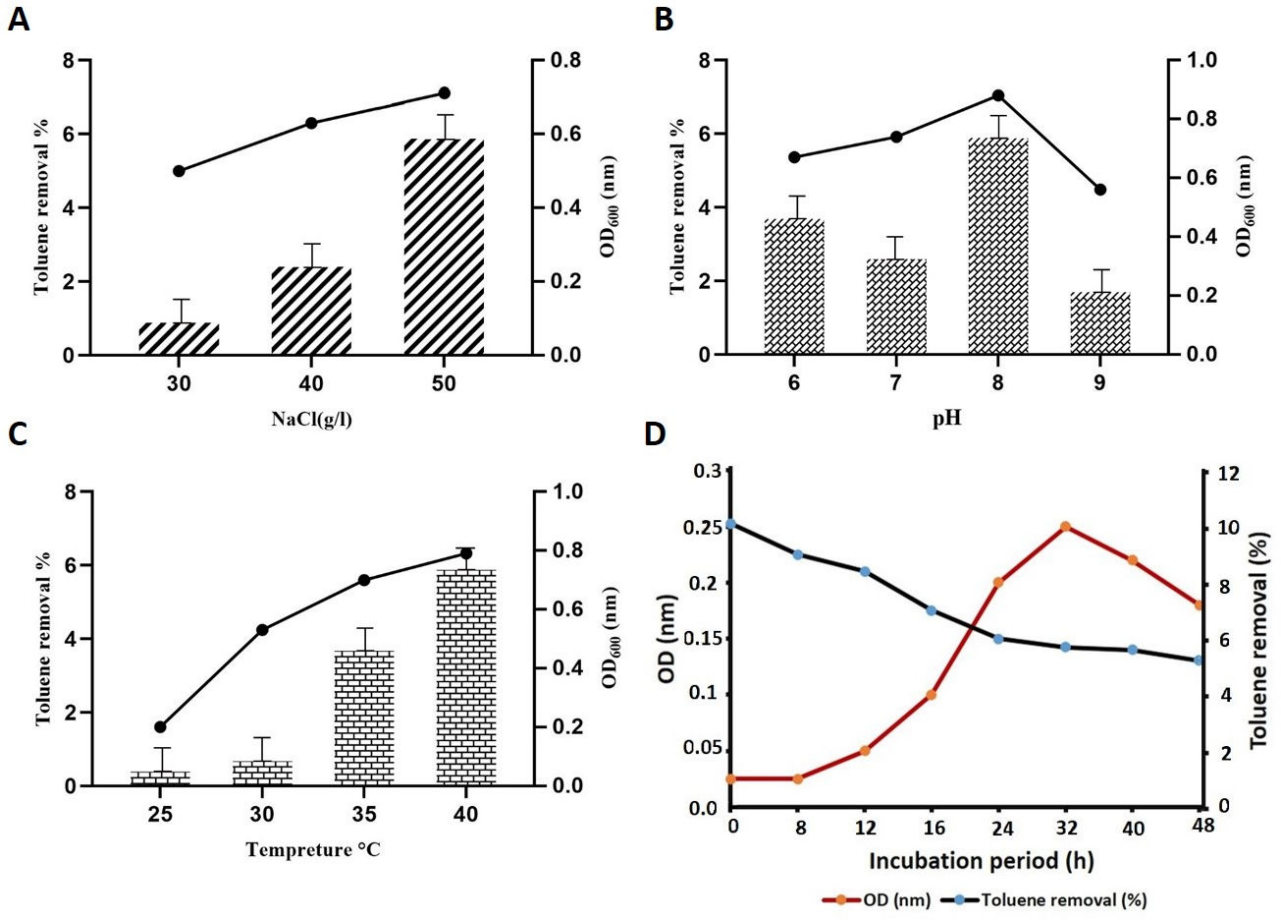
Effect of NaCl concentrations on (**A**) the toluene removal, (**B**) the optimal pH, and (**C**) the temperature, for the most toluene removal rate by Bacillus sp. GYRND102. (**D**) Growth curve, toluene removal by *Bacillus* sp. GYRND102 in the mineral medium supplemented with 10% toluene (vol/vol).

Figure 2D reveals GYRND102 strain growth in the presence of toluene (10% [vol/vol]) as the sole carbon and energy source. As shown, the isolate was in a lag phase up to 8 h after inoculation and grew gently. Then, the culture entered the log phase after 8 h and approximately stayed in this phase for 24 h. Figure 2D shows that the GYRND102 strain was efficiently able to remove toluene from the MSM. The highest rate of toluene removal also resulted after 24 h. Based on these results, there could be a direct relationship between the toluene consumption and bacterial growth rate.

### Effect of primary toluene concentration, NaCl, pH, and temperature on toluene removal

As seen in Fig. S2, the highest toluene removal rate, about 60% of the initial amount, occurred in the presence of 10% toluene (vol/vol) over 24 h. The results indicated that this strain can survive in the presence of high toluene concentrations as a sole source of carbon and energy.

Separate examinations were also performed to evaluate the toluene removal ability of the strain in a mineral salt medium containing 10% toluene (vol/vol) with different pH values, temperatures, and NaCl concentrations. As seen in Fig. 2, near 60% of the initial toluene (vol/vol) was removed in the NaCl concentration of 50 g/L, pH 8, at 40°C.

### Gas chromatography analysis of toluene degradation

Using gas chromatography–mass spectrometry (GC–MS), the metabolites resulting from the toluene degradation by the GYRND102 strain were qualitatively identified (Fig. S3A), which include acetate and 2-propanone (Fig. S3B). The *X*-axis shows that the retention time reflecting the elution of the compounds is between 2 and 26 min. The key peaks are at 5.219, 8.753, and 12.106 min, which are probably relative to larger or less volatile decomposition products. The *Y*-axis shows the intensity values (up to ~32 million), representing the relative concentrations of resulting compounds. The peaks at 12.106 and 5.219 min are the dominant peaks, which are the highest abundance and possibly reflect the main degradation products and the key intermediates, respectively. The peaks at <5 min represent volatile fragments such as acetaldehyde. The mid-range peaks (5–10 min) also likely indicate benzaldehyde (an oxidation product) or cresols. The late peaks (>10 min) represent benzoic acid. Therefore, the peaks at 5.219 and 12.106 min correspond to the retention times of benzaldehyde and benzoic acid (a carboxylated product), respectively. Moreover, the early peaks, such as 1.538, 1.678, and 2.939 min, could be acetone or 2-propanone. The mass spectrum (Fig. S3B) also shows the base peak at a mass-to-charge ratio (*m*/*z)* of 43 ([CH3CO]^+^) and the molecular ion peak at an *m*/*z* of 58 ([C3H6O]^+^) for acetone. Accordingly, it can be claimed that the toluene degradation route in strain GYRND102 is probably an oxidation pathway: toluene → benzyl alcohol → benzaldehyde → benzoic acid → CO_2_ (7, 42, 43). Also, the toluene breakdown pathway was investigated in the Kyoto Encyclopedia of Genes and Genomes (KEGG) database (PATHWAY: map00623). We identified the toluene degradation route (see Fig. 8) during which toluene converted to catechol and then to acetone and acetate via the meta-cleavage pathway.

### Characterization of the petrochemical effluent

Successful bioremediation of environmental pollutants is dependent on the ability to create and maintain conditions that ultimately increase the biodegradation of the pollutants (44). Here, the physicochemical parameters of a petrochemical waste effluent were measured to determine the effluent nature qualitatively, resulting in the efficient treatment of the effluent.

The physicochemical parameters of the Mahshahr petrochemical effluent are listed in Table 2. The effluent pH was 6.63, which is close to neutral pH. The electrical conductivity was 24,300, denoting the high salt content of the effluent. The chemical oxygen demand (COD) was 11,300 PPM, indicating the chemical (organic and inorganic) contents of the effluent. Furthermore, the effluent contained significant amounts of toluene, phosphorus (P), organic nitrogen, potassium (K), calcium (Ca), sulfate (SO₄²^−^), etc. that some microorganisms including bacteria can survive in the vicinity of these elements or by using aromatic hydrocarbons as energy and carbon sources.

**TABLE 2:** Physicochemical characteristics of Mahshahr petrochemical effluent^*a*^

Parameters	Mahshahr effluent
Turbidity	417 NTU
Electrical conductivity	24,300
Cl^−^	35,500 PPM
pH	6.63
COD	11,300 PPM
TOC	3,292 PPM
Sulfate	834,660 PPM
Calcium	120,000 PPM
Alkalinity	970,000 PPM
Sodium	4,500 PPM
Potassium	6.1 PPM
Ammonia nitrogen	1,803 PPM
Organic nitrogen	137 PPM
Total nitrogen	2,173 PPM
Nitrate	0
Nitrite	0
Total phosphorus	218 PPM
Lead	5.78 PPB
Chromium	521 PPB
Cadmium	220 PPB
Xylene	273,490 PPM
Toluene	32,491 PPM

^*a*^ NTU, nephelometric turbidity unit; TOC, total organic carbon.

### Effect of minerals, carbon source, and municipal wastewater on the bacterial activity in the petrochemical effluent

After defining suitable amounts of NaCl, temperature, and pH parameters for maximum toluene removal in the mineral salt medium, the effect of adding carbon source, minerals, and municipal wastewater on the bacterial activity in the petrochemical effluent was also investigated. The best concentration of each variable was obtained to increase the removal efficiency by the isolate.

The study of the bacterial activity in the petrochemical effluent with and without addition of minerals indicated meaningful variations in toluene removal efficiency. The ability of toluene degradation in the mineral-enriched effluent was investigated by the rate of reductions in COD and total organic carbon (TOC) contents. The cultures were incubated at pH 8 and 35°C to study degradation activity. The rate of TOC reduction in culture containing no minerals was about 13.6%, whereas with 25% and 50% of mineral salt medium, the TOC reduction efficiency increased by 7% and 10%, respectively. The COD-value reduction was also augmented with the addition of minerals. Consequently, the toluene removal efficiency by *GYRND102* in the presence of suitable minerals was meaningfully increased. In total, a decline of about 6%–24% in COD and TOC levels was observed after 24 and 48 h of incubation (Table S2).

Three different amounts of sucrose were utilized in separate experiments to probe the effect of carbon source on the COD and TOC reduction efficiency. As reported in Table S3, adding 0.5 g sucrose per 100 cc petrochemical effluent resulted in increasing the average reduction efficiency for COD and TOC by about 32.8% and 30.3% respectively, after 48 h incubation at 35°C. On average, augmenting the sucrose concentration by 1 g increased the COD and TOC removal efficiency by roughly 51%, whereas adding 2 g sucrose did not increase the COD and TOC reduction efficiency; instead, both decreased about 6% and 3%, respectively. Moreover, the control sample containing no external carbon source was also tested, which indicated an efficiency of about 16%. Eventually, based on the COD and TOC tests, it became clear that the studied bacterium was able to survive and degrade aromatic hydrocarbon available in the effluent in the presence of a specific concentration of sucrose (1 g).

Usually, in industrial wastewater, the amount of easily biodegradable organic matter (BOD) is low, and as a result, the growth of microorganisms and, consequently, the activity of total microorganisms in it is low and is not enough to remove hard degradable chemicals such as toluene. One of the ways to increase the BOD of industrial effluents is to mix industrial effluents with urban sewage and treat them with the presence of microorganisms that decompose industrial effluent compounds. For this purpose, in this study, Isfahan urban wastewater was added to the petrochemical wastewater, and its effect on COD and toluene removal was investigated.

The physicochemical parameters of the municipal wastewater are stated in Table S4. The pH of the urban wastewater was 7.15, indicating the near-neutral pH. Electrical conductivity was 1,146, which denotes salt content in the urban wastewater. The BOD value was 386 PPM, representing the presence of biologically degradable organic compounds in the urban sewerage. As reported in Table S5, the efficiency of the TOC reduction in culture containing a mixture of 50% petrochemical effluent and 50% municipal effluent was 24%. The COD reduction efficiency was also about 24% after adding municipal wastewater. In total, the results revealed about 23.6%–30.8% reduction in the COD and TOC levels after 48 h of incubation. Moreover, as seen in Table S5, the efficiencies of COD and TOC reduction were 30% and 28%, respectively, in the simultaneous presence of sucrose and municipal effluent, whereas the COD reduction efficiencies were 32.5% and 24% when they were used separately.

### Optimization of petrochemical effluent composition for bacterial activity by the Taguchi method

The Taguchi method was used to define the suitable conditions for efficient treatment of petrochemical effluent (Tables S19 and S20). According to the software design, orthogonal array L16 with 16 trials (Table S20) was used. After three replications of each experiment, Trial 11, which contained 5% petrochemical effluent, 0.5 g sucrose, 75% dry mineral salt medium (equivalent to 2.715 g), and 30% municipal effluent, showed the highest efficiency, with about 38.15% toluene removal (Table S20).

Conforming to software prediction, the optimum medium composition for bacterial growth and activity could be levels 1(0.5 g), 2 (30 cc), 1 (0.90527 g), and 3 (5 cc) for carbon source, municipal effluent (BOD), minerals, and petrochemical effluent, respectively. Experimental evaluation of this composition in three replicates resulted in a 41.55% decrease in COD after 48 h. Quantitative analysis of the medium composition by GC–MS before (Fig. S4A) and after (Fig. S4B) treatment with the bacterium strain revealed a significant reduction in organic compounds, especially toluene, which shows the COD and TOC reduction resulted from toluene decomposition by the desired bacterium. Consequently, this bacterium has a high ability to remove toluene using the minimum requirements and available materials.

### Genomic analysis of the GYRND102 strain

After confirming genomic DNA quality, reported in Table S6, whole-genome sequencing, applying the Illumina HiSeq 2500 sequencing platform, led to approximately 200× coverage for the genome. Collectively, DNA sequencing resulted in 7,906,458 raw reads sequenced in both directions with 150 bp in length and guanine-plus-cytosine (G+C) content of 38% (Table S7). The primary reads were subjected to filter low-quality scores (≤*Q*20) and a minimum read length of 40 bases, and to trim off five bases from 5′ ends to achieve proper conditions for downstream analysis (Fig. S6). The 7,330,704 clean reads were then *de novo* assembled, which eventuated in 41 contigs (>500 bp) with lengths ranging from 669 to 1,756,923 bp and an *N*50 size of 1,041,840 bp, ultimately resulting in a total length of 5,154,622 bp using a mixture of k-mers 81, 99, 113, 115, and 121 (Table S8). The 41 scaffolds, as the strain genome, were annotated to identify target genes and pathways. Moreover, evaluation of a genome entirety by BUSCO software showed that *Bacillus* sp. GYRND102 was 99.8% complete, indicating that most of the identified genes could be classified as complete and single copies (45).

### Phylogenetic analysis

**Fig 3:**
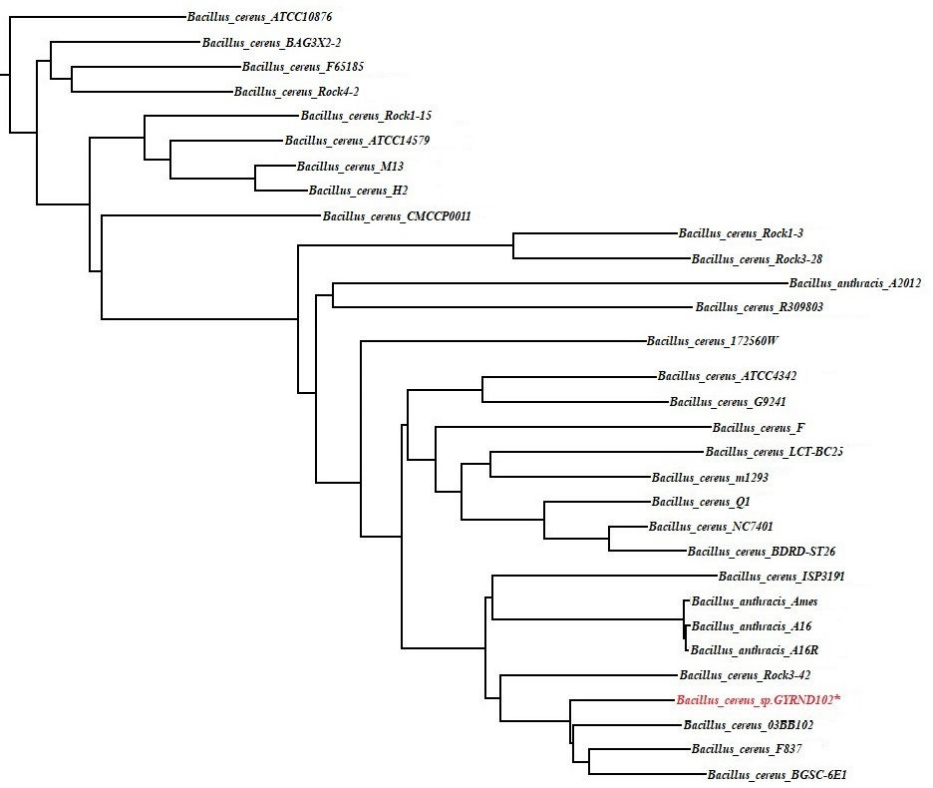
The CVTree of 31 bacteria. The tree is constructed with *K* = 6, based on all the amino acid sequences of the 30 strains related to the *Bacillus* genus.

**Fig 4:**
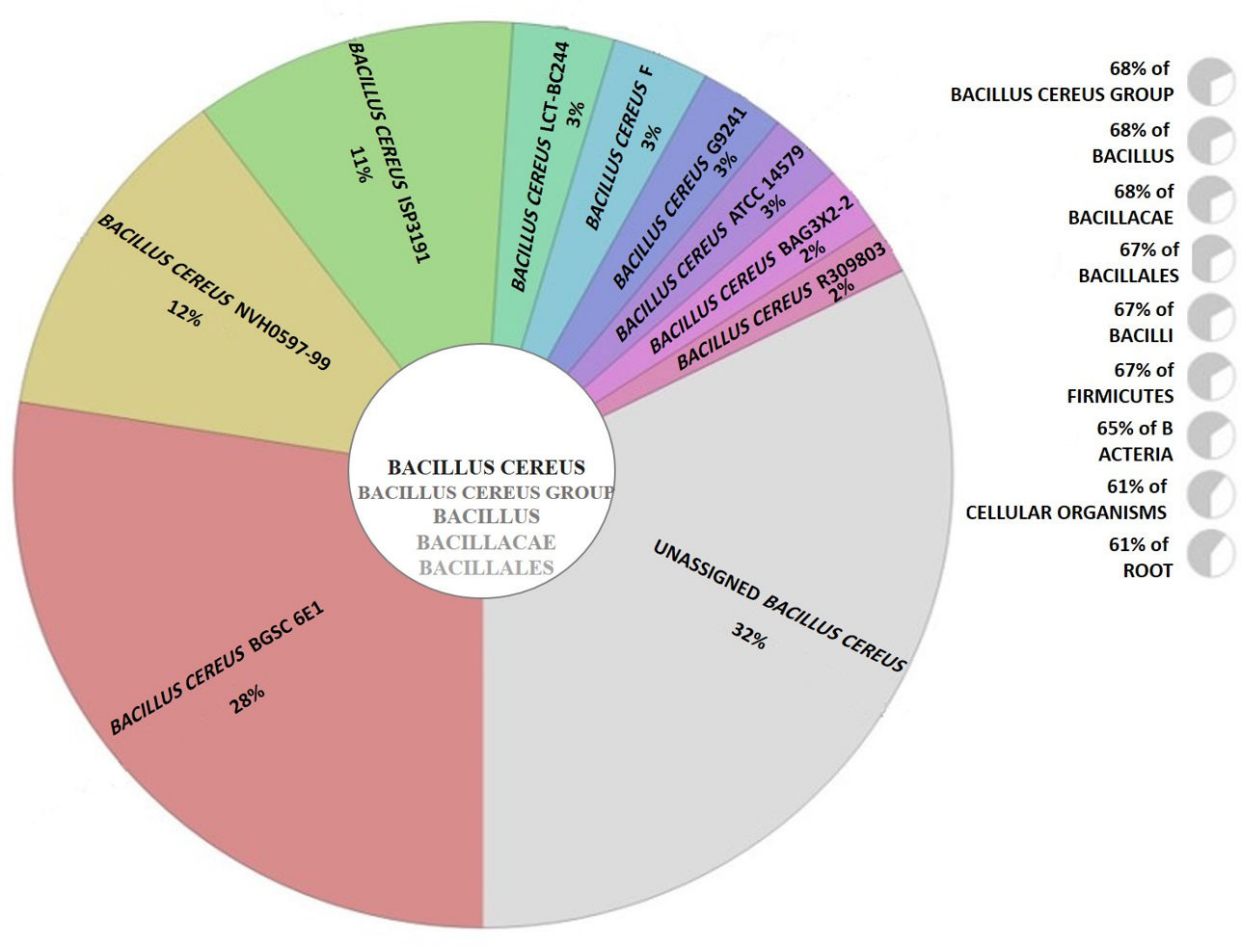
Taxonomic analysis of *Bacillus GYRND102* strain. Alignment of *GYRND102* reads to universal marker genes in Phylosift indicated *cereus* as the closest species.

The 16S rRNA sequence analysis, as a molecular marker, was subjected to identify the GYRND102 strain. The BLAST similarity investigation against the National Center for Biotechnology Information (NCBI) 16S rRNA gene database demonstrated that the G strain belonged to the genus *Bacillus* (Table S9), but no species was assigned as the closest species in this step. Also, the characterized sequence measuring 1,541 bp in length has been deposited in the NCBI database (accession no. KT751303.1). The phylogenetic relationship of *B*. *cereus* strain GYRND102 was more inferred based on analyzing housekeeping gene sequences, including *glpF*, *gmk*, *ilvD*, *pta*, *tpi*, *rpoB*, *gyrB*, *mdh*, *mbl*, and *mutS* genes (46, 47), leading to select *cereus* as the closest species (Table S10). Also, the phylogenetic analysis based on all the amino acid sequences (Fig. 3) indicated that the closest neighbor of strain GYRND102 is *B. cereus* strain 03BB102. In corollary, CVTree and PhyloSift (Fig. 4) analyses confirmed the multi-locus sequence typing (MLST) and mapping results, which indicate that the studied strain belongs to genus *Bacillus *and species *cereus*.

### Genome features of *B*. *cereus* GYRND102 and whole-genome comparison

**Fig 5:**
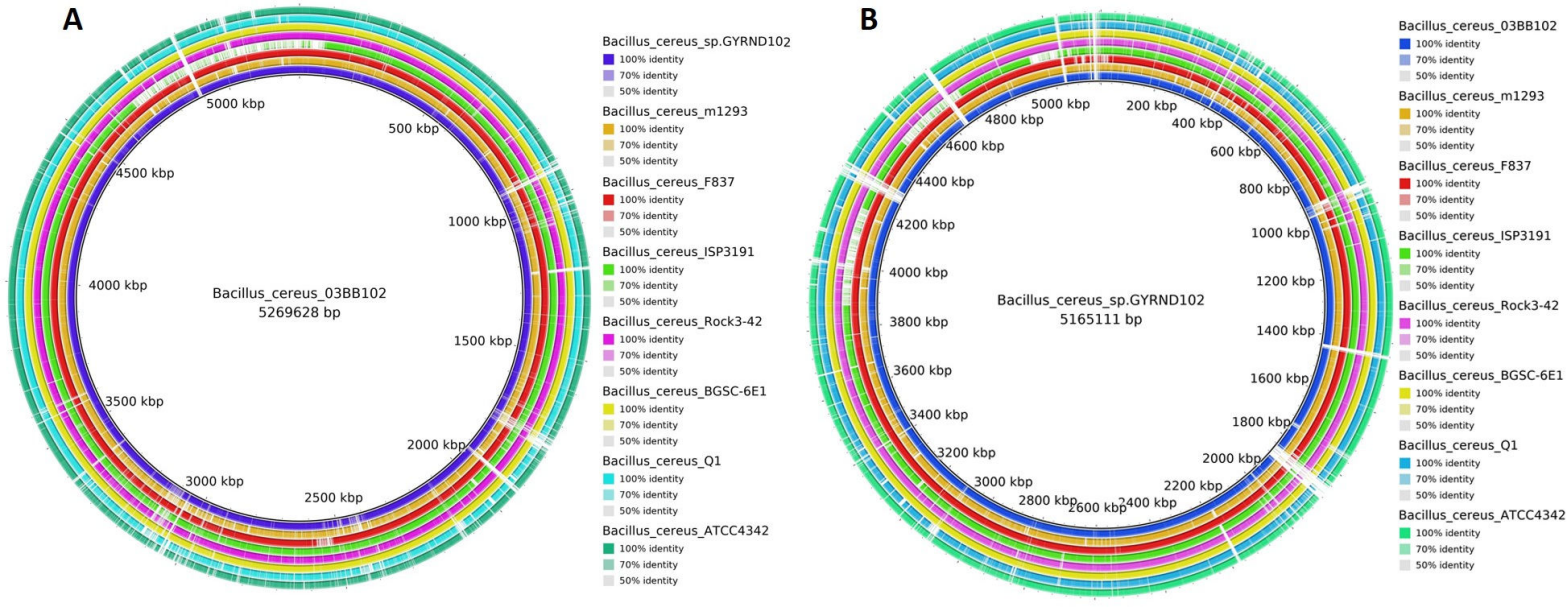
(**A**) Genome comparisons of *Bacillus cereus GYRND102* and other *B. cereus* strains against strain *03BB102* as the reference genome constructed using BRIG v.0.95. The circular graph illustrates the complete genome comparison of the strain *03BB102* with the other eight closely associated genomes of *B. cereus*. The internal black circle indicates the strain *03BB102* genome (reference genome), and each color intensity indicates the similarity rate of that strain with the reference genome. (**B**) Genomic comparison of the eight *B. cereus* strains against drafted *GYRND102* scaffold as genome reference. The interior black circle demonstrates the whole genome of the *GYRND102* strain, and the shade of each color illustrates the resemblances between each strain with the *GYRND102* strain.

The total length of the *B. cereus* GYRND102 genome was 5,154,622 bp (~5.1 Mbp) with a mean G+C content of 35.34%. This draft genome assembly contained a total of 5,355 genes, including 5,242 putative coding sequences (CDSs). In addition, a total of 113 RNA genes, including 16 rRNAs and 97 tRNAs, were anticipated in the chromosome sequence. The mapped read percentage to the eight relevant genomes was probed to detect the best genome as reference (Fig. S7). Eventually, we opted for the *Bacillus cereus* strain 03BB102 (GenBank: NZ_CP009318.1) as the closest strain with alignments of over 89.27% of the reads (Table S11). Extra genomic characteristics of *B. cereus* GYRND102 and eight intimately related genomes, including the genome size, distribution of the G+C content, total number of RNA genes, and predicted CDSs, are reported in Table 3; Fig. 5. Accordingly, the genomic sequence, genome size, total number of predicted CDSs, and total number of genes of the GYRND102 strain are similar but slightly smaller than those of the 03BB102, M1293*,* and BGSC-6E1 strains, with BGSC-6E1 possessing the largest genome size of ~5.7 Mb.

**TABLE 3:** Comparative genome analysis

Strain	Genome size (Mb)	GC %	Scaffolds	No. of tRNAs	No. of rRNAs	No. of proteins	No. of genes	GenBank accession number
GYRND102	5.165	35.34	41	97	16	5,242	5,355	JAJUXD000000000
03BB102	5.45	35.29	2	105	14	5,331	5,620	GCA_000022505.1
F837	5.29	35.40	3	105	12	5136	5,447	GCA_000239195.1
BGSC-6E1	5.73	35.00	1	105	14	5,137	5,620	GCA_000160915.1
ISP3191	5.37	35.30	4	76	15	5,273	5,487	GCA_000291135.1
Rock3-42	5.20	35.20	1	104	15	5,342	5,471	GCA_000161235.1
M1293	5.27	35.35	1	96	13	5,282	5,360	GCA_000003645.1
Q1	5.51	35.50	3	93	13	5,377	5,634	GCA_000013065.1
ATCC 4342^*a*^	5.31	35.37	2	111	17	5,312	5,444	GCA_000832845.1

^*a*^ ATCC, American Type Culture Collection.

**Fig 6:**
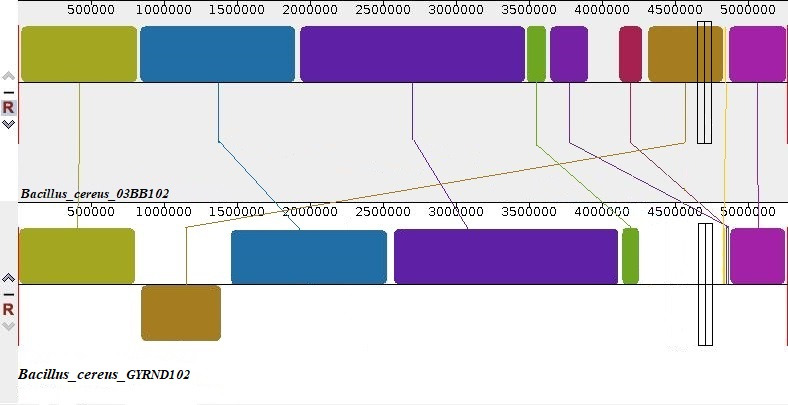
Genome alignment indicating syntenic blocks between *B. cereus 03bb102* strains (top) and the ordered genome sequence of the *GYRND102* strain (bottom).

By comparing the ordered genome assembly of the GYRND102 strain with the reference genome (strain 03BB102), the presence of nine collinear blocks was detected (Fig. 6). The chromosomal alignments encompassed various classes of events: inversion, deletion, and rearrangement regions. According to the existence of large homologous segments when most parts of the two genome sequences are mapped onto each other, it appears that the chromosomal alignments of these strains are nearly identical. Moreover, a segment between contigs 1 and 3 in the GYRND102 chromosomal scaffold approximately at 1.5 Mb illustrates inversion, showing various syntonical relationships to the reference chromosomal sequence. On the other hand, one deletion and also two translocation regions are seen in the GYRND102 chromosome between contigs 5 and 6 and contigs 5 and 9, respectively.

### Pan- and core-genome analyses of *B. cereus* GYRND102

**Fig 7:**
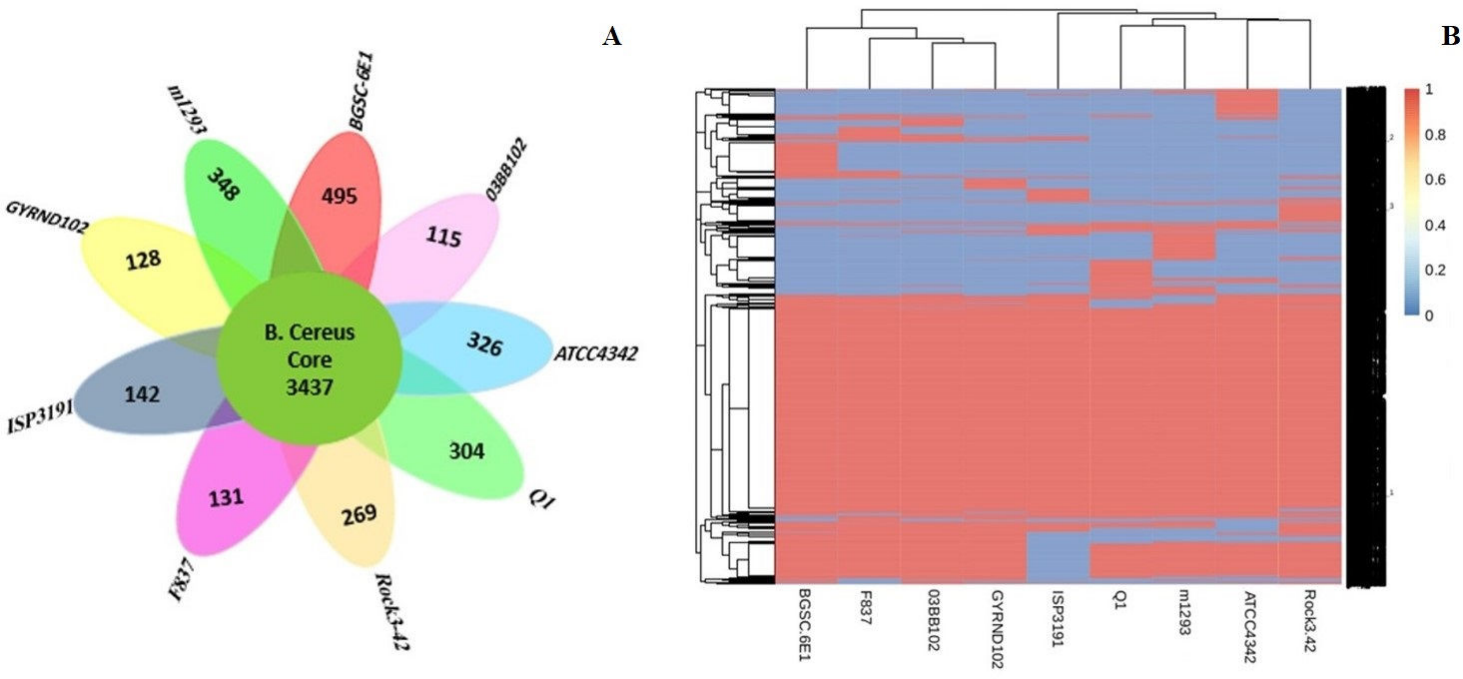
The core/pan-genome plot and the Venn diagram for the core-genome and strain-specific CDS of the *B. cereus* strains. (**A**) The number of unique genes for each strain of the *B. cereus* pan-genome analysis. (**B**) Pheatmap of *B. cereus* cloud and shell genes.

To analyze the GYRND102 and eight *B. cereus* strain pan-genomes in detail, the Roary pipeline was applied to compare the translated coding sequence set, continued via clustering of orthologous proteins and the representatives from each orthologous cluster and strain-particular CDS in the entire pan-genome. Of 8,523 orthologous proteins, 3,437 (40.3% of total CDS) are core genes—soft core and hard core—across all nine strains genomes. A total of 2,258 CDS (26.4%) accessory genes (shell and cloud) embrace as the accessory fraction, indicating the existence of unique genes to each genome. Strains 03BB102, F837, and ISP3191 encompass the smallest numbers of unique genes, with 115, 131, and 142 genes, respectively. Also, the highest number of specific genes belongs to the Q1, American Type Culture Collection (ATCC) 4342, M1293, and BGSC-6E1 strains, with 304, 326, 348, and 495 genes, respectively (Fig. 7). A total of 128 unique genes were also encoded by the target genome strain GYRND102, 86 of which have been anticipated to encode hypothetical proteins.

As reported in Table S12, all nine analyzed strains contain genes *catE*, xylH, *xylF*, *xylJ*, and *dmpG*, contributing to the degradation of aromatic compounds through the meta-cleavage route, but they possess no genes involved in the ortho-cleavage pathway. As reported in various studies, the initiation of sporulation in many species of *Bacillus* genus is regulated via the phosphorelay pathway, in which the *kinB*, *kapB*, *kinD*, *kinE*, *spo0F*, *spo0B*, *spo0A*, and *spo0M* genes play major roles (48). These regulatory genes have been found in the nine strains, excluding the *kinB* gene, which is just present in strain GYRND102. Also, the *kapB* and *spo0F* genes are absent in strain ISP3191. Furthermore, to identify the bacterial antibiotic resistance genes that eventually lead to bacterial survival in the presence of antibiotics, we appraised the clusters of orthologous CDS shared by *B. cereus* strain GYRND102 and its nine closest strains. After scrutinizing the core and pan-genomes, we found 16 genes, including *penP*, *catA*, *vat*, *ereA*_*B*, *vanW*, and *mprF* genes, associated with antibiotic resistance. Of those, we found the *ereA_B* and *aacC* genes only in strain GYRND102, whereas the *vanW*, *catA*, and *penP* genes in all nine strains were implicated in causing resistance to vancomycin, chloramphenicol, and ampicillin antibiotics, respectively. In addition, the *vat* gene, virginiamycin A acetyltransferase, in strain F837, and the *tetM* gene, tetracycline resistance protein, in strains BGSC-6E1, ISP3191, Q1, and M1293 were also found in our strain. Also, the *hxlR*, *mhqR_4*, and *hpxO_2* genes were identified only in the strain GYRND102, which acts as an HTH-type transcriptional activator, catechol-resistance regulon governor (49), and catalyzer of hydroxylation of urates to 5-hydroxyisourate (50), respectively. Moreover, we detected 31 known genes belonging to lyases, hydrolases, the toxin-antitoxin system, oxidoreductases, and transferases (Table S12).

### Intraspecific phylogeny analysis using core and pan-genomes

The phylogenetic relevance of *B. cereus* GYRND102 and eight other *B. cereus* strains was shown separately based upon the pan- and core genomes. As seen in Fig. S5A, analysis based on the accessory genes clustered *B. cereus* GYRND102 in a separate clade, genetically close to strains 03BB102 and Rock3-42. Moreover, this analysis illustrated a distant phylogenetic relation between strains G, Q1, and M1293, which were clustered together in the same clade close to strain GYRND102. A phylogenetic tree (Fig. S5B) was also constructed using the sequences of 3,437 conserved genes (core genome), which revealed the clustering of the GYRND102 and 03BB102 strains in the same clade, genetically close to the F837 genome and in the backward step close to the BGSC-6E1 genome.

### Pathway analysis

To study gene function, genes in whole genome are assigned with a KEGG Orthology (KO) identifier, which is followed by interpreting these K numbers (51). Genome annotation resulted in the prediction of approximate catabolic, biochemical, and biosynthetic routes. Also, the metabolic classification indicated genes involved in each metabolism group (Fig. S8). Interpretation of genomic data identified various genes related to aromatic compound degradation pathways, as well as genes involved in a two-component regulatory system and secretion systems, flagellar assembly, and antibiotic resistance (Tables S14 to S16).

### Aromatic compound degradation pathways

By analyzing the KO numbers assigned in the GYRND102 genome, we found about 65 candidate genes probably implicated in the degradation of the aromatic compounds, including toluene, benzoate, benzene, aminobenzoate, chloroalkane, and chloroalkene. We also found genes coding xenobiotic monooxygenases and flavin-containing monooxygenases, cytochrome P450 monooxygenase (accession no. NZ_ JAJUXD010000002, NODE2 [236353-236856]), alcohol dehydrogenase (accession no. NZ_JAJUXD010000003, NODE3 [284732-285934 and 244828-245865]), flavin monoamine oxidase (accession no. NZ_JAJUXD010000003, NODE3 [478522-479985 and 567671-569107]), as well as genes coding flavocytochrome P450 (accession no. NZ JAJUXD010000017.1, NODE17 [6163-7398]), anthranilate 3-monooxygenase (FAD) or 4-hydroxyphenylacetate 3-monooxygenase (accession no. NZ_ JAJUXD010000003, NODE3 [435549–437021]), flavin-dependent oxidoreductase (accession no. NZ_JAJUXD010000001, NODE1 [633800-634124], and accession no. NZ_ JAJUXD010000003, NODE3_ [552768-553823]), alkanesulfonate monooxygenase (accession no. NZ_ JAJUXD010000001, NODE_1 [215108-216220]), and alkanal monooxygenase alpha chain or flavin-dependent oxidoreductase (accession no. NZ JAJUXD010000003, NODE3 [552768-553823]), which play a crucial role in converting aromatic compounds into catechol (Table S14). Notably, the *GYRND102* genome possesses just the catechol 2, 3-dioxygenase (*catE*) gene (accession no. NZ_JAJUXD010000001, NODE_1 [869979-870917]), which guides the catechol to the meta-cleavage pathway for more degradation (Table S14). Additionally, genes encoding ring-cleaving dioxygenase enzymes (accession no. NZ_ JAJUXD010000002, NODE2_ [190212-191189 and 189223-190167]) were also found in the *GYRND102* genome. As presented in Table S13, the *GYRND102* genome encompasses all genes implicated in the meta-cleavage pathway and no genes related to the ortho-cleavage route. Furthermore, we could not identify a complete group of genes associated with the degradation of other aromatic compounds.

### Secretion systems

**Fig 8:**
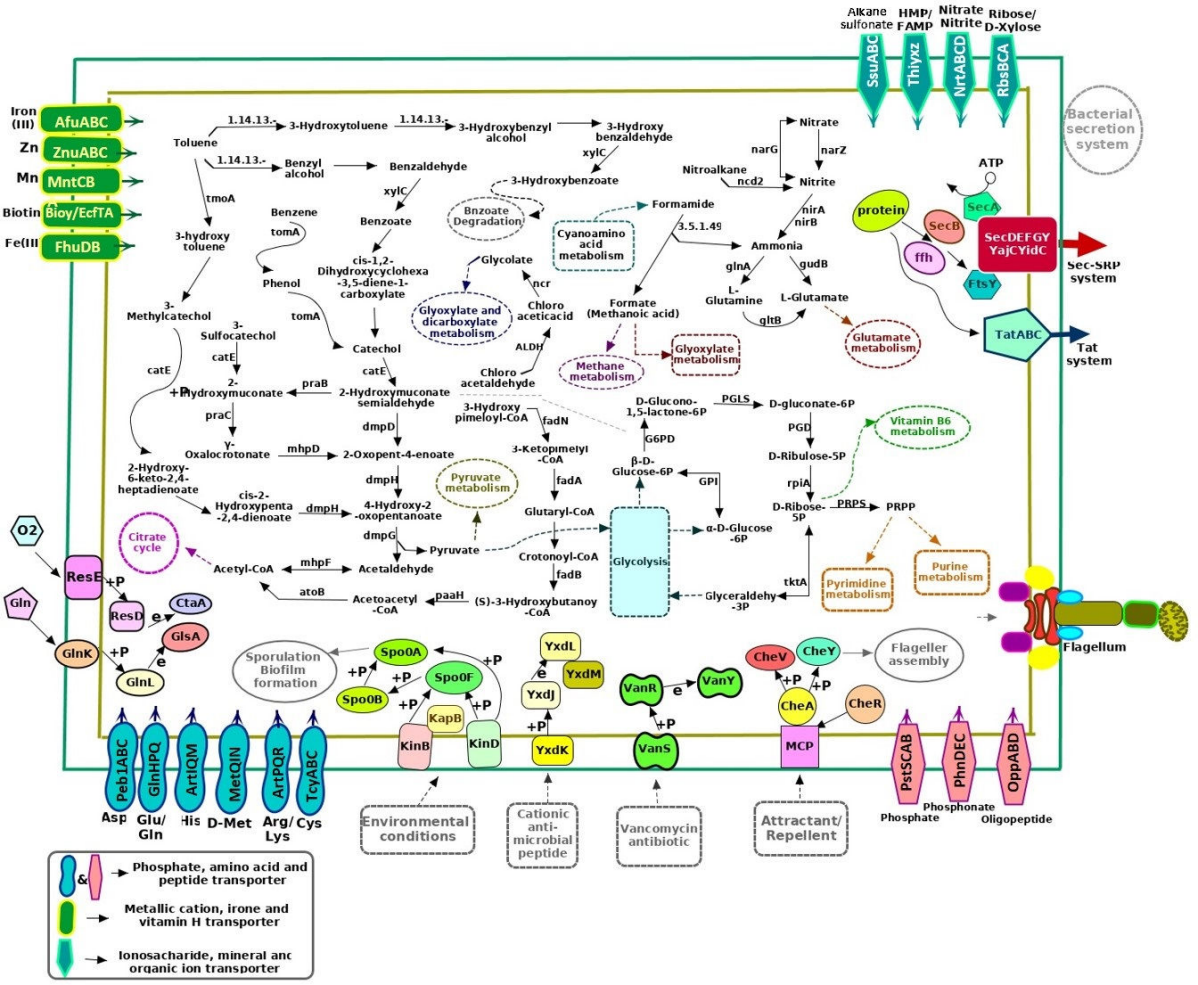
Schematic overview of metabolic pathways of *B. cereus GYRND102*. The portrayed routes were recognized relying on the *GYRND102* genomic data, showing the strain possesses genes consistent with the decomposing ability of several aromatic compounds. The strain also carries a complete set of genes that contributed to carbohydrate metabolic pathways involving the Krebs (tricarboxylic acid) cycle, glycolysis/gluconeogenesis, the pentose phosphate pathway, and pyruvate metabolism. The latter contains the genes involved in the flagellar assembly system, as well as the secretion systems, including Tat and Sec-SRP systems. Furthermore, multiple genes are present in this strain relating to nitrogen metabolism, two-component regulatory systems, and the ABC transporter system.

By the genome annotation, nearly 15 genes were found involved in the general secretory pathway (Sec-SRP), twin-arginine translocation (Tat) systems, and flagellar assembly. Approximately all genes coding protein subunits related to the Tat and Sec-SRP and flagellar assembly systems were identified in the genome (Table S15; Fig. 8), except *secB*, encoding pre-protein translocase subunit SecB, and *tatE*, encoding sec-independent translocase protein *TatE* genes.

### Antibiotic resistance in the GYRND102 genome

By annotating the genome, approximately 42 candidate genes involved in antibiotic resistance routes were identified (Table S16). These genes encode virginiamycin acetyltransferases (52), beta-lactamases (53), erythromycin esterase (54), and chloramphenicol acetyltransferases (52), enzymes that take part in the antibiotic decomposition of virginiamycin, ampicillin, erythromycin, and chloramphenicol, respectively. The *vanW* gene encoding the vancomycin B-type resistance protein was also found, which plays a role in inactivating vancomycin and conferring resistance to this antibiotic (55).

### Quorum sensing

According to data reported in Table S18, 87 genes implicated in two-component regulatory systems were found in the GYRND102 genome, such as the ResE-ResD, PhoR-PhoP, VicK-VicR, Spo0A-P, and CheA-CheYBV systems, as well as GlnK-GlnL and YufL/YufM two-component systems, which were activated in response to glutamine and malate, respectively. The genes related to the antibiotic-sensing system named LiaRS two-component system were also identified (Fig. 8). Moreover, the *GYRND102* genome possesses nearly 60 genes associated with the quorum sensing process, probably acting either directly or indirectly through cellular metabolism (Table S17). Of those, the *nprR*, *plcR*, and *nprX* genes encode proteins that are intracellular QS receptors. NprR is detected as the activator of a neutral protease, and it is also related to sporulation and extracellular protease activity in some strains of the *B. cereus* group (56). In addition, the *oppA* gene encodes the oligopeptide-binding protein, which is a periplasmic protein in gram-negative bacteria and is responsible for the peptide attraction from the exterior medium that exists in the stain (57). Also, the *yidC* gene codes another membrane protein, YidC, implicated in the insertion and folding of multitude membrane proteins, being indispensable for cell viability (58).

## DISCUSSION

Since a major portion of hydrocarbon pollutants accumulate in the environment and pose a serious threat to human and environmental health, the application of efficient, environmentally friendly, and regionally adaptable approaches is inevitable to remediate contamination (8). In this study, the *Bacillus cereus* GYRND102 strain, a novel toluene-degrading strain, was isolated from Isfahan petrochemical soil. Here, we investigated the optimal conditions to use this strain to treat an industrial effluent containing various aromatic compounds, including toluene. The results indicate that the remediation of effluent pollutants can be accelerated by using active indigenous microorganisms and ameliorating the environmental conditions, including nutrient addition. Moreover, we sequenced, *de novo* assembled, and analyzed the genome of the *B. cereus* GYRND102 strain, with genomic data confirming and supporting observations of its ability to decompose some aromatic compounds, including toluene. The high genomic propinquity among *B. cereus* GYRND102 and eight formerly sequenced *B. cereus* strains, especially strains 03BB102, F837, and BGSC-6E1, was indicated through comparative genomic analysis. Besides, a constructed phylogenetic tree based on the accessory genes illustrated *B. cereus* GYRND102 in a separate clade, genetically close to the o3BB102 and *Rock3-42* strains. Also, a phylogenetic tree based on the core genome clustered *B. cereus* GYRND102 and o3BB102 in a single clade (Fig. S5B), genetically close to the BGSC-6E1 and F837 strains, which indicates their evolution from a common ancestor. Furthermore, analysis of the pan-/core genomes brought forward some clues on the particular genes implicated in antibiotic resistance, sporulation, and flagellar assembly among the studied strains of *B. cereus*.

According to the results, the GYRND102 strain was able to produce considerable amounts of peroxidase enzymes such as catalase, which can assist in the bioremediation of aromatic pollutants (59). Interestingly, the GYRND102 strain showed a strong ability to decompose toluene, achieving a maximum removal efficiency of 41.55% in aerobic conditions. The highest toluene removal rate, about 60%, materialized by target strain on an initial 10% toluene (vol/vol) and at an optical density (OD) of over 0.25. It can be concluded that the toluene consumption by the GYRND102 strain is directly correlated with its growth rate. Hence, less bacterial growth can pose a reduction in toluene biodegradation.

According to researchers, “There is a direct relationship between a decrease in biodegradation and a decrease in temperature” (60). As reported, the highest toluene removal rate and microbial growth resulted at a temperature of 40°C. The hydrocarbon metabolism rate is enhanced to a maximum at the temperature ranging from 30°C to 40°C, above which prompts the augmentation of the membrane toxicity of hydrocarbons (22). The pH, toluene concentration, and salinity are other factors affecting the bacterial growth and biodegradation rate. Based on the results, greater growth rates were observed in the low toluene concentrations (≤10% vol/vol) than in the higher primary concentrations.

Moreover, the results of this study were not consistent with the results reported by Wang et al. (61) and Afrouzossadat et al. (22), which presented that the hydrocarbon biodegradation rates that declined with enhancing salinity may be due to the nature of different microorganisms used in these studies. *Bacillus* sp. GYRND102 removed a higher percentage of toluene at NaCl 50 g/L, whereas the percentage of toluene removal was lower at concentrations of 30 and 40 g/L. Since GYRND102 has been isolated from a salty environment (Table 2) and growth in the presence of NaCl 50 g/L is a halotolerant (relatively halophilic) bacterium, it can grow and perform its biological activities better in a relatively salty medium (62–65). On the other hand, most regions of Iran are classified as salty arid and semiarid regions, and most petrochemical industries are also located in these regions. Consequently, toluene-degrading bacteria with the capability to tolerate and grow in these environments can greatly contribute to the degradation of toluene in extreme environments (66). The research by Nicholson and Fathepure indicated the ability of the halotolerant and halophilic bacteria to rapidly break down BTEX in the presence of 145 g/L (2.5 M) NaCl under aerobic conditions (67). Li et al. reported that the initial organic wastewater concentration of 2,000 mg/L, 20 mL bacterial inoculum, cultivation time of 72 h, and salt concentration of 20% resulted in a 66.4% reduction in COD (68). Also, Mainka et al. mentioned that the use of halophiles to degrade the aromatics in saline environments has shown high removal efficiencies for the tested contaminants (69).

The neutral pH is preferred by the majority of microorganisms (70), but it is mentioned that the oxidation rates of hydrocarbons fluctuate at the pH range of 6–8 (71). The maximum growth was observed at a pH of 8 by our strain (Fig. 2B); consequently, the target bacterium can grow better at alkaline pH.

Despite the toluene toxicity to living organisms, microorganisms, especially bacteria, can degrade and eliminate toluene due to their high metabolic power and fast adaptation to their environment (72, 73). Hamzah et al. (74) have reported ODs of 0.1 and 0.04 in the medium containing 0.5% toluene (vol/vol) for *Pseudomonas* sp. and *Bacillus* sp., respectively. Also, Pratheesh and Jayachandran (75) observed an OD of 0.13 for a strain of *Pseudomonas* in the presence of 0.02% toluene. They also stated that the further increase in effluent concentration impacts bacterial growth negatively due to toxicity (76). In the present study, the ability of the GYRND102 strain to remove toluene was investigated in MSM containing 1%–20% toluene (0.108–2.7 M). The results indicated that this strain was highly capable of tolerating toluene concentrations and removing 6% out of 10% toluene (Fig. 2D; Fig. S2). Wang et al. (61) also reported that toluene-degrading strains, *Exiguobacterium gaetbuli* and *Bacillus mojavensis*, were able to tolerate 5%–20% toluene concentrations (77). Also, Heydarnezhad et al. reported that the toluene degradation and cell growth of *Bacillus cereus* ATHH39 reached 64.11% and OD_600_ = 0.69, respectively, by adjusting the temperature, pH, and toluene concentration to 33.16°C, 6.72, and 824.15 mg/L respectively (78).

Research has shown that adjusting carbon to nitrogen and carbon to phosphorus ratios stimulates and increases the biodegradation of toluene and other specific hydrocarbons (60). Using boosters is effective in removing pollutants, and optimizing the amount of the additives has to be considered according to the climatic conditions. So, we used sucrose, municipal effluent, and MSM as the primary available carbon, phosphorus, nitrogen, and trace salts sources needed by bacteria. Based on the results, the COD reduction rate, without adding any variable parameters, was between 7.4% and 14.7%. Removal efficiency was increased through microbial stimulation by adding sucrose, dry mineral salt medium, and municipal effluent (increasing the microbial population and BOD) by about 33%, 23%, and 33%, respectively. Using the Taguchi L16 orthogonal array showed the best removal efficiency of about 38% in three replications for a concentration of 5% petrochemical effluent, 2.715 g minerals, 0.5 g/L sucrose, and 30% municipal effluent (Table S20). The removal efficiency improved to about 42% in optimum conditions suggested by experimental design (0.5 g/L sucrose, 30% municipal effluent, 0.905 g minerals, and 2% petrochemical effluent). Accordingly, a simultaneous increase of nutrients and suitable microbial population inoculation plays an effective role in removing toluene from a petrochemical effluent. Remarkably, the results of this study are consistent with the results reported by other researchers such as Cheney et al. (79), Bento et al. (80), and Sarkar et al. (81). Additionally, genetic manipulation of the aromatic compound degradation metabolic pathway of the strain can lead to increased potential for aromatic compound degradation and use in bioremediation of polluted environments.

In general, during aerobic bioremediation of aromatic compounds, the resulting activated molecules undergo ring cleavage reactions and are more processed to generate molecules that ultimately enter the tricarboxylic acid cycle (82). As reported, the ring cleavage reactions proceed through the ortho- or meta-cleavage pathways (4, 83). The *dmpB*, *dmpC*, *dmpD*, and *dmpH* genes code the main enzymes catalyzing the meta-cleavage route (84). The ortho-cleavage pathway is also carried out by the *catRBCA* gene cluster converting catechols to 3-oxoadipate intermediates (85). In this study, results showed that the GYRND102 strain contains all genes implicated in the meta-cleavage pathway (Table S13), but no genes contributed to the ortho-cleavage route. According to the pan-genome analysis, the genes for the ortho-cleavage route are absent in all nine strains, whereas almost all genes involved in the meta-cleavage pathway are available in all strains except the *dmpC* gene, which is present only in the GYRND102 strain.

The GYRND102 strain contains enzyme-coding genes associated with pathways degrading some aromatic compounds, namely, toluene, benzoate, benzene, xylene, and chloroalkane. Remarkably, we could not find the genes encoding enzymes that catalyze the initial reactions transforming the mentioned aromatic compounds to catechol, such as genes encoding toluene-4-monooxygenase and xylene monooxygenase (14). As presented in the literature, various monooxygenases have been introduced as key enzymes to catalyze the aromatic ring hydroxylation at variant positions (82, 86). Kirchner et al. reported that “The phenol ortho-hydroxylation to catechol has been catalyzed by a phenol hydroxylase from the Flavin-dependent monooxygenase family in *Bacillus thermoglucosidasius A7*” (86). As reported by other researchers, the primary steps, converting aromatic ring to catechol, are carried out by an ammonia monooxygenase in *Nitrosomonas europaea* (87), toluene dioxygenase in *Pseudomonas putida* (14), cytochrome P450 monooxygenase in *Sphingomonas* (88), and as well as flavoprotein monooxygenases in *Pseudomonas pickettii PKO1* to hydrolyze carbolic acid (89). Since the GYRND102 strain contains flavin-dependent monooxygenases, xenobiotic monooxygenases, anthranilate 3-monooxygenase (FAD), 4-hydroxyphenylacetate 3-monooxygenase, alkanal monooxygenase alpha chain, and flavocytochrome P450, as well as aromatic ring-cleaving dioxygenase enzymes (Table S14), we can claim that the GYRND102 strain likely catabolizes the initial reactions of an aromatic ring breakdown to form the corresponding catechol using these enzymes. On the other hand, fragments of the GYRND102 genome may not have been sequenced at the DNA sequencing stage. Thus, maybe some genes in question are located in the gaps, so we could not identify them in the final genomic scaffolds. In addition, the existence of the genes engaged in the aromatic compound breakdown on the genome of the GYRND102 strain makes it suitable to employ in the bioremediation of aromatic compounds.

The genome size of *B. cereus* GYRND102 strain (5.165 Mbp) is smaller than the genome size of strains such as 03BB102 (5.45 Mbp) and BGSC-6E1 (5.73 Mbp) (Fig. 6; Table 3). The genome size of varied strains of a bacterial species can vary due to several factors, including horizontal gene transfer, mutational changes such as deletions, and repetitive sequences. By environmental adaptations, strains of various environments may have developed distinguished sets of genes (including toluene degradation in this strain) needed for survival in respective habitats or lost non-essential genes over time, leading to variations in genome size and content. In total, these factors contribute to the observed variation in genomic sizes among bacterial strains, reflecting the evolutionary pressures that each strain has undergone throughout history (90–94). Also, Cole et al. discovered amounts of gene deletion when comparing Leprosy *bacillus* with ancestral bacteria (95). Besides, the genomic size of 5–6 Mbp can be ascribed to the variegation of chromosomally encoded genes (93). It was reported that the recognition of genes encoding proteins involved in the capture of trace elements, in addition to those involved in invasion, is important because they are mostly related to redox processes, which are vital for microbial survival and growth, particularly under stress conditions (96, 97). In addition, the genome sequence of the *B. cereus* GYRND102 strain is whole-genome shotgun sequencing (JAJUXD000000000), and in comparison with the reference strain (*03BB102*), there are some gaps which may be the cause of smaller genome sizes.

Taxonomic identification is considered as the main component of microbial community studies (98). The taxonomy is done based on different gene sequences, in which the 16S rRNA-based classification has always been the most comprehensive and widespread case in microbiology today (99). The phylogeny study based on the 16S rRNA gene sequence indicated that the GYRND102 strain is owned by the genus *Bacillus*. However, this traditional molecular phylogeny uses only small subunit ribosomal RNA sequences (100). Also, there are several classification contradictions between various 16S databases; hence, many improvements are still required (101). MLST, a technique for typing of multiple loci, discriminates microbial strains by comparing DNA sequences of several housekeeping genes at the genus and species levels (102); accordingly, it is used to conduct more detailed studies of phylogenetic relationships (103). The sequence scrutiny of 10 housekeeping genes, including *glpF*, *gmk*, *ilvD*, *pta*, *tpi*, *rpoB*, *gyrB*, *mdh*, *mbl*, and *mutS*, represented that the *GYRND102* strain belongs to the genus *Bacillus* and the species *cereus*. Several new phylogenetic analyses have been carried out based on sequences of all proteins or genes (100). Hence, the phylogenetic correlation of the GYRND102 strain and 30 other strains was also scrutinized through the alignment of protein sequences of all strains. In the corollary, the GYRND102 strain is owned by the genus *Bacillus* and the species *cereus* (Fig. 3), which was corroborated by the phylogenetic studies at the read level (Fig. 4; Fig. S7). Consequently, the GYRND102 strain is considered a *cereus* species being owned by the genus *Bacillus*.

Production of an enzyme is one of the mechanisms by which bacteria can protect themselves from antibiotics (104). As previously reported, some enzymes, including beta-lactamases (53), chloramphenicol acetyltransferases, virginiamycin acetyltransferases (52), and erythromycin esterase (54), contribute to the decomposition of ampicillin, chloramphenicol, virginiamycin, and erythromycin antibiotics, respectively. Vancomycin B-type resistance protein is also encoded by the *vanW* gene, which inactivates vancomycin (55). We identified nearly 42 genes, mainly genes *penP*, *catA*, *vat*, *ereA_B*, *vanW*, and *mprF*, which can contribute to the resistance of the GYRND102 strain (Table S16) to the mentioned antibiotics. Also, based on the pan-genome analysis, the *ereA_B* and *aacC* genes were only available in the GYRND102 strain, whereas the *vanW*, *penP*, and *catA* genes were present in all nine strains, engaged in creating resistance to vancomycin, ampicillin, and chloramphenicol, respectively.

Two-component regulatory systems organize the main stimulus-response coupling mechanisms. These systems permit bacteria to sense and react to changes in environmental conditions (105). More general, bacterial two-component systems govern a stunning array of functions such as antibiotic resistance, cell division, and metabolite fixation and usage, responding to environmental stress and sporulation, as well as monitoring the bacterial flagellar motor (106). We detected 87 genes in the studied strain that are most likely implicated in the two-component regulatory systems (Table S18). Of those, notable two-component systems include VanS_B_-VanR_B_, KinB-Spo0F, YxdJK, LiaRS (YvqCE), and methyl-accepting chemotaxis protein (MCP)-CheWAY. Evers et al. (55) have described that transcription of vancomycin resistance genes, *VanS* and *VanR*, is activated in *Enterococcus faecalis* V583 by the VanR_B_–VanS_B_ two-component regulatory system. In addition, the LiaSR two-component system is utilized by multiple *Streptococcus* spp. inclusive of *Streptococcus mutans*, *Streptococcus pneumoniae*, and *Streptococcus agalactiae* in response to cell-envelope stressors like polymyxin B, vancomycin, β-lactams, and bacitracin, leading to the activation of genes engaged in cell wall maintenance (107). Also, Pietiäinen et al. studied the stress response of *Bacillus subtilis* to membrane-active cationic antimicrobial peptides and indicated that this reaction is governed by the YxdJK two-component regulatory system activated by a special activator named LL-37. The YxdJK controls the expression of the YxdLM ABC transporter as well (108). Furthermore, KinA and KinB were known to be potentially responsible for phosphorylating the Spo0F response regulator and subsequently initiating sporulation in *Bacillus subtilis* (48). Strikingly, the phosphoryl group is conveyed to Spo0B, and eventually to Spo0A; then, sporulation is triggered when the Spo0A∼P level reaches a critical level (109). Moreover, MCPs sense environmental stimuli and transfer signals to the flagellar motor via a phosphorelay two-component signaling cascade (110). Sensing a signal by the MCPs augments CheA autophosphorylation. The phosphoryl group from CheA is transmitted to the response regulator CheY. Then phosphorylated CheY protein binds to a switch on the flagellar motors to trigger their rotation (111) toward chemical compounds, including aromatic hydrocarbons (112). On the other hand, the CheA-CheYBV regulatory system has been reported in *Salmonella* Typhimurium, contributing in reaction to environmental physical and chemical stimuli (113). Since the *GYRND102* strain can grow on some aromatic hydrocarbons as lonely carbon and energy sources and also contains the main resistance genes to multiple antibiotics and cationic antimicrobial peptides, it is probably able to respond to environmental chemical stimulus regulated by the CheA-CheYBV system and antibiotic resistance governed by the VanSB-VanRB, YxdJK, and LiaRS systems.

As mentioned, the *B. cereus* GYRND102 strain showed resistance to some antibiotics, in addition to the ability to break down the toluene. The relationship between toluene decomposition and antibiotic resistance is an emerging study area indicating how bacteria can adapt to environmental stresses, including contaminants such as toluene and antibiotics. The existence of both pollutants and antibiotics may drive the co-selection of resistant strains. It has been proposed that there is a probable relation between organic solvent tolerance and decreased sensitivity to other types of chemicals, including antibiotics (114). Santos et al. put forward a link between the notable solute-stress forbearance of mycobacteria and their environmental tenacity (115). It has been stated that the specific lipid composition of mycobacteria (outermost layer, cell wall, and conventional plasma membrane) has been linked to resistance to antibiotics and disinfectants (114). Also, it has been suggested that environmental conditions may affect bacterial susceptibility to antibiotics through the promotion of stress responses, leading to an alteration of gene expression patterns and cell physiology (116, 117). Additionally, Pacífico et al. presented that *Mycobacterium vaccae* cells generated the required fatty acids for maintaining the fluidity level requisite for cell growth and survival during adaptation to both methyl tert-butyl ether (MTBE) and ethanol. They reported that cells exposed to and adapted to organic solvents revealed lower forbearance to the antimicrobial agents, levofloxacin and teicoplanin, than non-adapted ones. However, MTBE-adapted cells were able to tolerate higher concentrations of both antibiotics than ethanol-adapted cells (114). This interplay between hydrocarbon decomposition and antibiotic resistance may indicate an intricate interaction formed by environmental conditions and evolutionary pressures. However, more studies involving varied classes of antibiotics and solvents are needed to thoroughly comprehend the relation between antibiotic resistance and biodegradability of pollutants and solvents, including toluene. The comprehension of this relationship could lay out insights into bioremediation strategies.

### Conclusion

Using bacteria or its enzymes to break down pollutant compounds assists in overcoming the constraints and troubles related to other common chemical procedures (5). In this study, we proposed to isolate a novel bacterial strain, *Bacillus cereus* GYRND102, from the environment for its potential to decompose toluene. This indigenous strain could be used as a biological substance for the biodegradation of toluene. Here, the Taguchi method was employed to explore the effects of process factors on the amount of toluene removal in the Mahshahr petrochemical effluent. Adding nutrients and inoculating the appropriate microbial population simultaneously had a very effective role in removing toluene from the effluent. In addition, analysis of the *Bacillus cereus* GYRND102 strain genome confirmed its toluene degradation capability through identifying multiple potential genes related to the degradation of aromatic compounds through the meta-cleavage pathway. Moreover, phylogenetic analysis based on the housekeeping genes and all amino acid sequences indicates that the GYRND102 strain belongs to *Bacillus cereus*. The whole-genome comparison of *B. cereus* GYRND102 with eight other closely associated *cereus* strains revealed a total of 3,437 and 2,258 CDSs as the core-conserved and accessory genes, respectively. Furthermore, the GYRND102 strain contains a set of antibiotic resistance genes coding the β-lactamase, virginiamycin A acetyltransferase, and chloramphenicol acetyltransferase enzymes. This study aims to present a global view of the GYRND102 strain, which will certainly assist in providing further insight into unraveling the complicated mechanisms that GYRND102 and other similar bacteria may be used to remove aromatic compounds. However, the exact detection and elucidation of the decomposition mechanism of aromatic pollutants and how they are regulated need more research.

## MATERIALS AND METHODS

### Chemicals and reagents

All chemicals and reagents utilized in this study were of analytical grade and were prepared according to standard methods for the examination of water and wastewater published by the American Public Health Association press (76).

### Sample collection

Bacteria capable of degrading aromatic compounds under saline conditions are more likely to be obtained from environments with long-term exposure to pollutants. Therefore, in this project, six samples were randomly collected from several saline sites contaminated with toluene under standard conditions. Three soil samples were collected from the Isfahan Petrochemical zone, and one sample was obtained from the Morcheh Khort industrial site around the Fidar factory. One sample was also collected from petrochemical wastewater from Isfahan and another one from the saline water of a lake near Meymeh County in Isfahan Province.

### Media and culture conditions

Laboratory experiments were designed to identify strains capable of using toluene as the sole carbon and energy source and of growing on saline media. The samples were inoculated in a mineral medium after being transferred to the laboratory. The samples were screened in two stages. In the initial screening, soil or effluent and saline water samples were inoculated in mineral medium (0.2 g toluene, 1.5 g K_2_HPO_4_.3H_2_O, 4 g NaNo_3_, 0.5 g Na_2_HPO_4_, 0.2 g MgSO_4_.7H_2_O, 0.01 g CaCl_2_, 0.001 g FeSO_4_.7H_2_O, and 30 g NaCl per liter) incubated at 37°C on a shaker at 200 rpm. Grown bacteria in the mineral medium were streaked onto Luria–Bertani (LB) agar medium (10 g peptone, 5 g yeast extract, and 10 g NaCl, 15 g agar per liter). The resulting separate various bacterial colonies were used for secondary screening. In the secondary screening, the resulting isolates were inoculated into liquid mineral medium (0.4 g toluene, 1.5 g K_2_HPO_4_.3H_2_O, 4 g NaNo_3_, 0.5 g Na_2_HPO_4_, 0.2 g MgSO_4_.7H_2_O, 0.01 g CaCl_2_, 0.001 g FeSO_4_.7H_2_O, and 30 g NaCl per liter). In this step, experiments were designed to identify strains with greater ability to use toluene and grow on saline media. To prepare fresh colonies, bacteria were streaked onto LB agar medium and incubated at 37°C. Also, once in pure culture, they were stored in 30% glycerol at −70°C.

### Morphology and biochemical tests

In the morphological experiments, bacterial macroscopic facial features were examined on the solid culture medium. Gram staining was done by the Hucker method (118).

The isolated strain was characterized by various biochemical tests, namely, oxidase (119), catalase (120, 121), starch hydrolysis (122), indole (123), methyl red and Voges–Proskauer (123), nitrate reduction (123), Simmons citrate (124), gelatin hydrolysis (125), and triple sugar iron tests (126). The results were compared with *Bergey’s Manual of Determinative Bacteriology* (127).

### Growth rate and toluene removal assay

To evaluate the bacterial growth rate, bacteria were cultured in 10 mL of mineral salt medium containing different concentrations of toluene, 1%–20% (vol/vol), incubating at 35°C with shaking at 180 rpm for 24 h. Bacterial growth was assayed spectrophotometrically by measuring the OD of the culture sample at 600 nm. The toluene removal rate was measured for a concentration of toluene, which most bacteria grew well in. After determining the best concentration, the bacteria were first cultured in 10 mL of mineral salt medium containing 10% toluene (vol/vol) (the best concentration) and incubated at 35°C at 180 rpm. After 24 h, 1 mL n-hexane, as toluene solvent, was added to each medium and mixed. Then, the media were centrifuged for 15 min at 6,000 rpm. Using a spectrophotometer, the optical density of their supernatants was measured at 282 nm (59). A sample with no bacterial inoculation was also applied as a control. Ultimately, the amount of removed toluene was determined, and accordingly, the bacteria with more ability to remove toluene were selected for subsequent studies. Also, the growth curve of the selected isolate cultured in the mentioned medium (10% toluene) was drawn at an assigned time interval (6 h).

### Effect of temperature, pH, and NaCl on cell growth

The effect of NaCl in a range of 30, 40, and 50 g/L on the growth and ability of isolates in degrading toluene was studied. Furthermore, the effect of temperature and pH on the selected strain growth was examined, as well as its degradation ability using mineral salt medium (NaCl 50 g/L) completed with 10% toluene (vol/vol), the strain inoculation, and by incubating in a range of different temperatures (25°C, 30°C, 35°C, and 40°C) and different pH values (6, 7, 8, and 9). Uninoculated tubes served as controls. The opacity of the media was measured by a spectrophotometer at a specific wavelength (600 nm).

### GC analysis of bacterial metabolites

To identify the secondary metabolites, GC–MS analysis was accomplished by a GC–MS system (Agilent 7000 Series Triple Quad Column, 30 m × 250 µm × 0.25 µm). The extraction process was carried out according to Thermo Fisher Scientific (Instruction BSTFA, 1255.1, 2008) with organic solvents. The derivatization process was accomplished with BSTFA [N, O-Bis(trimethylsilyl)trifluoroacetamide] to enhance thermal stability and volatility. Afterward, the prepared sample was analyzed by the GC–MS system, which was briefly described (128). The extracts (1 µL aliquots) were injected into a capillary column by programmable temperature vaporization (PTV) injection in the splitless mode. The temperature of the PTV was adjusted at 70°C during injection, but 0.6 min after injection, the temperature was increased to 300°C with a rate of 2°C/s and held at the same temperature for 20 min. The initial temperature for the GC oven was set at 70°C, which was raised 5 min after injection to 320°C at a rate of 5°C/min. By adjusting the pressure, the helium flow, as a carrier gas, was retained constant at a flow rate of 1.7 mL/min. Detection was obtained by MS detection in electron impact mode and full scan monitoring mode with an *m*/*z* range of 15–800. The temperatures of the ion source and quadrupole were adjusted to 250°C and 200°C, respectively. The bacteria were cultured in 20 mL of mineral salt medium containing 10% toluene (vol/vol), incubated at 35°C at 180 rpm. After 24 h, 2 mL hexane, as a toluene solvent, was added to each medium and mixed. Then, the media were centrifuged for 15 min at 6,000 rpm, and the supernatants were separated.

### Petrochemical effluent characterization

To investigate the treatability of the Mahshahr petrochemical effluent, the effluent physicochemical parameters were measured. Accordingly, the petrochemical effluent characterized for various physicochemical parameters, comprising electrical conductivity (EC) (by EC meter; Hach Company, Loveland, CO, USA), pH, turbidity, COD, TOC, potassium, total nitrogen, and phosphorus, and 14 other ones reported in Table 2, was measured, following the standard analytical method (76).

### COD measurement

The solutions of COD assay were prepared by the closed reflux colorimetric method. Briefly, 2.6 g K_2_Cr_2_O_7_, 42 mL H_2_SO_4_, and 8.33 g HgSO_4_ were mixed followed by diluting to a volume of 250 mL of deionized water. To prepare the catalyst solution, 5.06 g Ag_2_SO_4_ was added to 500 mL of H_2_SO_4_ and stirred for 48 h to completely dissolve the Ag_2_SO_4_ in the solution. To analyze the COD, the catalyst solution (3.5 mL) was added to the digestion solution (1.5 mL) in steel and screw-cap test tubes (10 mL) and then allowed to cool to room temperature. To analyze the COD through the miniaturized method, the catalyst solution in a volume of 560 µL was added to 240 µL of the digestion solution in screw-cap test tubes and permitted to cool to room temperature. Effluent samples were added to the miniaturized and standard COD ampules and incubated for 2 h at 150°C in a dry incubator (Hach Company). After cooling the COD tubes to ambient temperature, COD levels were detected by measuring the absorbance of the digested assay solution at *λ* = 282 nm by a Shimadzu UV-visible spectrophotometer (129).

### Effect of minerals on the bacterial growth and activity in the petrochemical effluent

To study the effect of minerals on the growth of the strain in the petrochemical effluent, various amounts of mineral medium (1.5 g K_2_HPO_4_.3H_2_O, 3 g NaNo_3_, 0.5 g Na_2_HPO_4_, 0.2 g MgSO_4_.7H_2_O, 0.01 g CaCl_2_, 0.001 g FeSO_4_.7H_2_O, and 30 g NaCl per liter) were added to the effluent, then it was incubated at pH 8 and 35°C with shaking at 180 rpm for 48 h. A closed reflux colorimetric method was applied to analyze the COD, using potassium dichromate (K_2_Cr_2_O_7_) as a standard solution, and total organic carbon was assessed using a Sievers* 5310 C Series TOC analyzer (76).

### Effect of carbon source on the bacterial growth and activity in the petrochemical effluent

To evaluate the effect of carbon source on the strain growth in the effluent, different amounts of sucrose (0.5, 1.0, and 2.0 g) were added to each of the petrochemical effluents with a dilution of 0.01 as an external carbon source and a supplier of the initial growth of bacteria. These cultures were then incubated at pH 8 and 35°C under persistent shaking (180 rpm) for 48 h and passed through a 0.45 µm filter paper. The COD and TOC tests were carried out using the methods previously described (76).

### The municipal wastewater impact as a BOD supplier in petrochemical effluent treatment

The urban wastewater as a BOD supplier was characterized to explore its impact on enhancing the bacterial performance in petrochemical wastewater treatment. Hence, some physicochemical parameters, including pH, electrical conductivity, turbidity, BOD, COD, and TOC, were identified as described earlier (76). Following that, the entrance effluent of Shahin Shahr wastewater treatment system was added to the petrochemical effluent (at a dilution of 0.01) at a ratio of 1:2. After incubating at 35°C, pH 8, with shaking (180 rpm) for 48 h, and passing through a 0.45 µm filter paper, the COD and TOC were measured. Moreover, the simultaneous effect of municipal effluent, carbon source, and minerals on petrochemical effluent treatment was investigated. To obtain this, 0.5 g sucrose (dissolved in 100 mL) and municipal effluent at the ratio of 1:2 and minerals were added to the petrochemical effluent. After incubating under the mentioned conditions for 48 h, the COD and TOC were measured for the solution at the 24th and 48th h.

### Statistical analysis

In this study, four independent variables consisting of carbon source, minerals, petrochemical effluent, and municipal effluent at four levels were used. The Taguchi method was utilized to designate the suitable experiential conditions for achieving the highest toluene removal efficiency, which proposed the orthogonal array L16. Also, statistical software, such as Design-Expert 6.0.6 (130) and MINITAB (131), was employed to analyze the experimental data. The designed experimental runs in this perusal are also presented in Table S20. Data are given as the mean of three replicates (mean ± standard deviation).

### GC analysis of the petrochemical effluent

GC–MS was performed, as already expressed, for quantitative analysis of mixtures of organic compounds existent in the effluent of the petrochemical refinery in two stages: studying the bacterial function on removing pollutants before and after optimizing the process parameters.

### DNA extraction

Genomic DNA was extracted in accordance with the following procedures:

1. A single colony of the bacterium grown on LB agar medium was inoculated into LB broth (10 g peptone, 10 g NaCl, and 5 g yeast extract per liter) and incubated for 20 h at 37°C with shaking at 180 rpm.
2. Cells were harvested by centrifugation at 6,000 rpm for 15 min, and the supernatant was discarded.
3. Buffer A (1.5 mL; Tris-HCl 100 mM, EDTA 5 mM, and NaCl 200 mM), along with SDS 10% (0.8 mL), was added to the bacteria.
4. After inverting for 5 min, debris was pelleted by centrifugation at 6,000 rpm for 15 min.
5. The supernatant (600–700 µL) was transferred to a 2.0 mL microcentrifuge tube, and 1.5 mL of a mixture of phenol, chloroform, and isoamyl alcohol (at a ratio of 25:24:1) was added.
6. The tube was inverted slowly and centrifuged at 4,000 rpm for 5 min.
7. The supernatant (600–700 µL) was gently transferred to a fresh 2.0 mL microcentrifuge tube, then an equal volume of isopropanol was added.
8. The tube was inverted to mix and then centrifuged at 10,000 rpm for for 5 min.
9. After discarding the supernatant, 0.1 mL of 70% ethanol was added to the pellet, then the pellet was gently resuspended.
10. DNA was precipitated for 5 min at 10,000 rpm.
11. After discarding the supernatant, the tube was incubated for 5–10 min at 55°C in a heat block and then left at room temperature for 30 min to evaporate the alcohol.
12. Distilled water (0.05 mL) was added to the tube to dissolve the DNA. DNA was stored at −20°C for extended periods or 4°C until use.

DNA quality was confirmed via agarose gel electrophoresis. DNA quantity was also estimated using the NanoDrop spectrophotometer (Thermo Scientific Nano Drop 2000c, USA). To further appraise the genomic DNA quality, PCR was performed for the 16S rRNA gene using universal primers, which are B1 (AGAGTTTGATCCTGGCTTAG) and B2 (TAAGGAGGTGATCCAGC) (132, 133). Then, the 16S rRNA gene sequence was applied to determine the genus and possible species of bacterium via the alignment against NCBI 16S ribosomal RNA sequence database.

### Genome sequencing and assembly

Genome of the bacterium was sequenced (BGI Tech Co., Ltd, Shenzhen, China) using the Illumina HiSeq 2500 Sequencing Platform (Illumina Inc., San Diego, CA). The DNA sample was applied to produce a paired-end sequencing library using the Nextera DNA Library Preparation Kit based on the manufacturer’s protocol. The quality of raw reads (score of 40) was detected via the FastQC v.0.11.9 (https://www.bioinformatics.babraham.ac.uk/projects/fastqc/) and followed by filtering and quality trimming by Trimmomatic v.0.40 tools (http://www.usadellab.org/cms/index.php?page=trimmomatic). Furthermore, the cleaned reads (screened high-quality sequences) were *de novo* assembled in various k-mer lengths and combinations using the SPAdes v.3.9.0 program (134). QUAST v.5.0.2 (135) was then used to assess the quality of each assembled genome. Finally, the combination of k-mers 81, 99, 113, 115, and 121 was chosen as the ultimate and optimized parameter to create the best scaffold. Then, the high-quality selected scaffolds were ordered by abacas v.1.3.1 tool (http://abacas.sourceforge.net) (136) using the closest genome reference. Moreover, the BUSCO v.3 tool (https://gitlab.com/ezlab/busco) was applied to evaluate the quality of the *de novo* assembly.

### Annotation

The protein-coding sequences of the bacterial genome sequence were predicted using two annotation tools: Prokka v.1.11 (137) and Prodigal v.2.6.3 (http://compbio.ornl.gov/prodigal/). Some servers, including RAST (http://rast.nmpdr.org/) and BlastKOALA v.2.1 (138), were used to annotate the genome of the ordered assembled genome and to identify existing genes. Using the FeatureExtract 1.2L Server (http://www.cbs.dtu.dk/services/FeatureExtract/) led to filtering and reporting the general features of the genome, such as rRNA and tRNA. Subsequently, the annotated genes were examined to determine which pathways they were involved in.

### Pathway analysis

The pathfinding approaches were applied to analyze the predicted and annotated gene sequences to find out the existence of certain metabolic pathways. Hence, the relevant pathways were entirely identified through manual inspection of the designated gene functions based on comparison with the KEGG routes (139). The identified biological processes were graphically illustrated using PathVisio v.3.2.0 (http://www.pathvisio.org/).

### Phylogenetic analysis

The genus and species of the strain were identified by using the MLST approach (102) based on the chosen housekeeping genes analysis. To identify the bacterial species, Phylosift software (http://edhar.genomecenter.ucdavis.edu/~koadman/phylosift/phylosift_latest.tar.bz2) was also applied to create phylogenetic models. The PhyloSift reference database is accessible independent of the software at this address: http://edhar.genomecenter.ucdavis.edu/~koadman/phylosift_markers (140). Finally, a phylogenetic tree was reconstructed based on the alignment of amino acid sequences of the 30 chosen strains, implementing the bootstrap method available in CVTree v.3.0 (http://tlife.fudan.edu.cn/cvtree/cvtree3/).

### Whole-genome mapping and core/pan-genome analyses

To identify the closest species genome, all reads sequences were mapped to all the accessible reference genomes using Bowtie2 v.2.3.5 software (141). We employed Mauve v.2.4.0 (http://darlinglab.org/mauve/download.html) software to peruse genome rearrangements in the bacterium draft genome and closely related bacteria. So, the multiple genome alignment of the whole-genome sequences of *B. cereus* strain *03BB102* (GCA_000022505.1) and these strain genomic scaffolds was constructed and illustrated by the progressive Mauve program available in the Mauve tool. Pan- and core-genome analyses of *B. cereus* strains comprising this strain and its eight close strains using Roary v.3.11.2 (http://sanger-pathogens.github.io/Roary) were accomplished to recognize the core- and strain-specific genes. Similarly, a phylogenetic tree was constructed implementing the Roary pipeline, on the basis of the presence/absence of core and unique genes.

### Comparative genomic analysis

Using the NCBI databases, the genome of the strain and eight closely related genomes were compared for several genomic features, including the genome size, the gene number, the GC contents, and the tRNA and rRNA numbers of the participating strains. To give a general view of whole-genome comparisons, we used the BRIG v.0.95 application (http://brig.sourceforge.net/). Accordingly, the eight intimately related genomes of the *B. cereus* strain, namely, 03BB102, F837, BGSC-6E1, Rock3-42, ISP3191, M1293, Q1, and ATCC 4342, were aligned and compared with the strain draft scaffold assembly. Moreover, pairwise genome alignment of the strain chromosomal scaffold with the eight mentioned strains was also carried out and represented.

## Data Availability

The data acquired during this study are presented in this paper and its supplemental files. Moreover, additional information about the whole-genome sequencing project of *Bacillus cereus* GYRND102 strain is deposited at https://www.ncbi.nlm.nih.gov with BioProject code PRJNA792958 and under the accession number JAJUXD000000000.

## SUPPLEMENTAL MATERIAL

Supplemental figures and tables spectrum.02892-24-s0001.pdf Figures S1 to S8; Tables S1 to S19.

ASM does not own the copyrights to Supplemental Material that may be linked to, or accessed through, an article. The authors have granted ASM a non-exclusive, world-wide license to publish the Supplemental Material files. Please contact the corresponding author directly for reuse.
