# Supplementary material for: Comprehensive genomic analysis of a novel Bacillus cereus decomposing toluene potentially applicable in bioremediation
Source: Microbiol Spectr. 2025 Dec 1;14(1):e02892-24. doi: 10.1128/spectrum.02892-24 (PMC12772314; doi:10.1128/spectrum.02892-24)
Supplement: Supplemental figures and tables — Figures S1 to S8; Tables S1 to S19. [file spectrum.02892-24-s0001.pdf]

Microbiology Spectrum

Supporting Information

Comprehensive genomic analysis of a novel *Bacillus cereus* decomposing toluene potentially applicable in bioremediation

Maryam Safari[1,3], Samira Ghahroodian[1], Mohamad vahid Abyarazimi[1], Samira Rahmaniyan[1], Fatemeh Heydaryan[1] & Marzieh Rezaei[1,4], Bagher Yakhchali[1,2*]

1. Department of Biology, Faculty of Science, Nour Danesh Institute of Higher Education, Isfahan Province, Meymeh, Danesh Blvd, I. R, Iran
2. Institute of Industrial and Environmental Biotechnology, National Institute of Genetic Engineering and Biotechnology (NIGEB), Tehran, I. R. Iran
3. Current address: Department of Biotechnology, Iranian Research Organization for Science and Technology (IROST), Tehran, I. R. Iran
4. Current address: Department of cell & Molecular Biology, Faculty of Science and Biotechnology, University of Isfahan, Isfahan, I. R. Iran

*Corresponding Author:

Bagher Yakhchali, Prof.
Institute of Industrial and Environmental Biotechnology, National Institute of Genetic Engineering and Biotechnology, Tehran, Iran.

E-mail: Bahar@nigeb.ac.ir

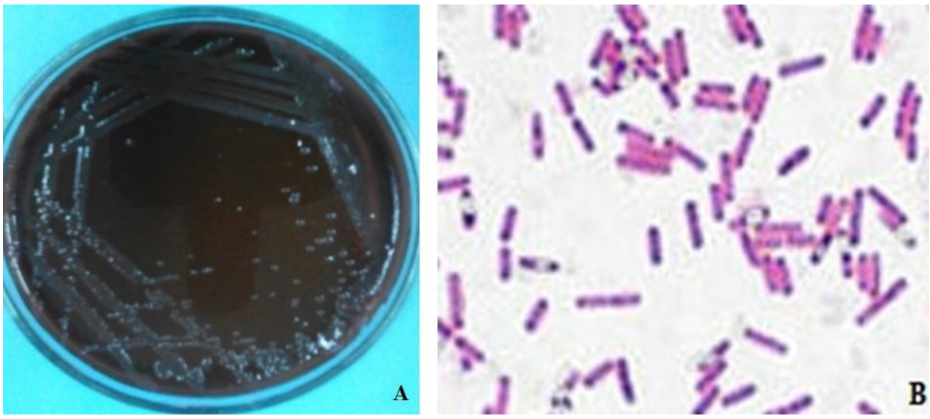

Figure S1. Morphological characters and gram stain for bacterium isolate A (GYRND102) isolated from Isfahan Petrochemical (soil), Isfahan, Iran.

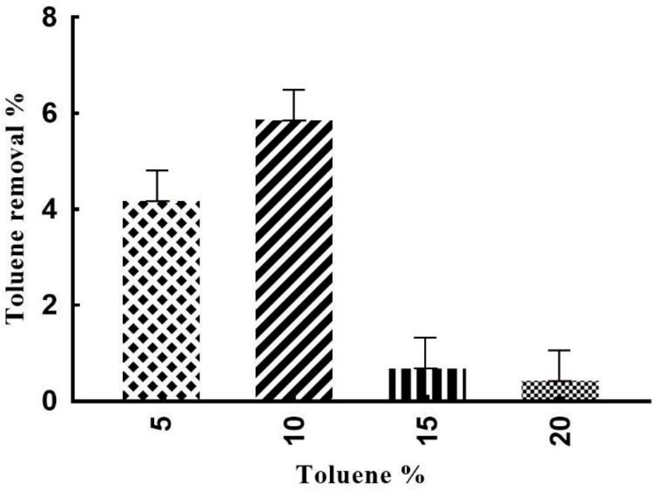

Figure S2. Effect of primary toluene concentrations on toluene removal by *Bacillus cereus GYRND102*

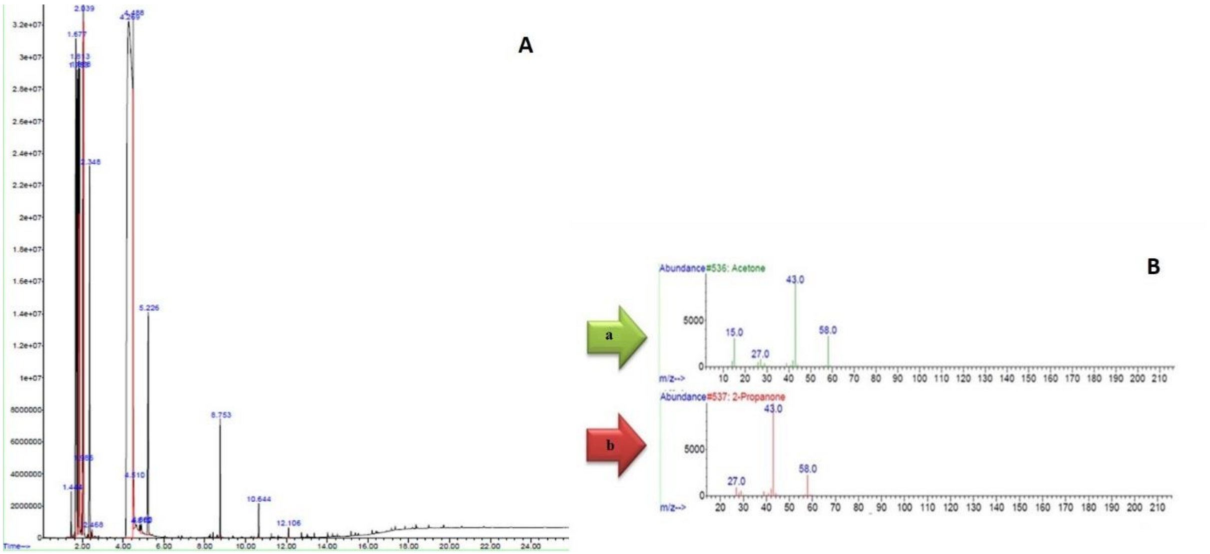

Figure S3. A) GC Analysis of the toluene breakdown by *Bacillus cereus*. *GYRND102*. **B)** The chromatography results of the produced metabolites through the toluene degradation (a: Acetone, b: 2-propanone)

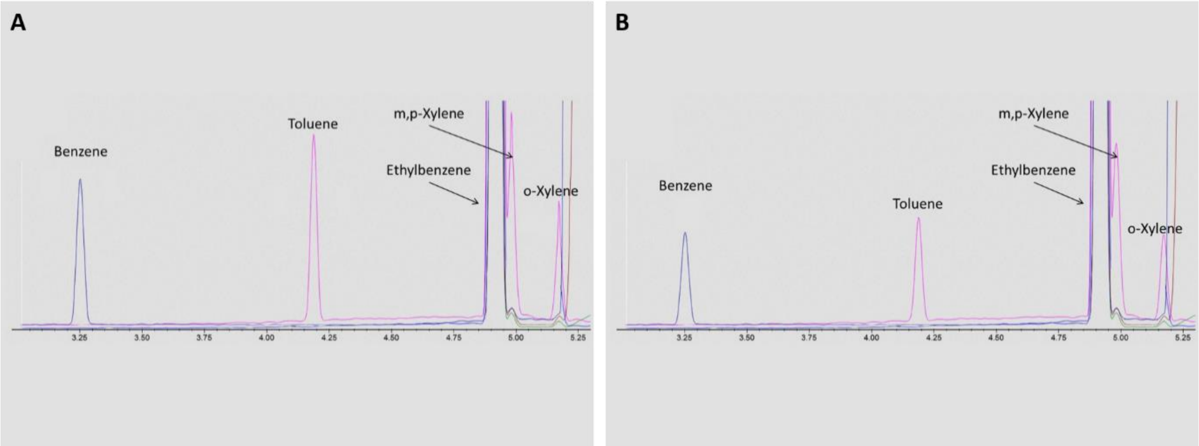

Figure S4. (A) Gas chromatography analysis of petrochemical effluent, **(B)** Gas chromatography analysis of petrochemical effluent after bacterial culture under optimal experimental conditions.

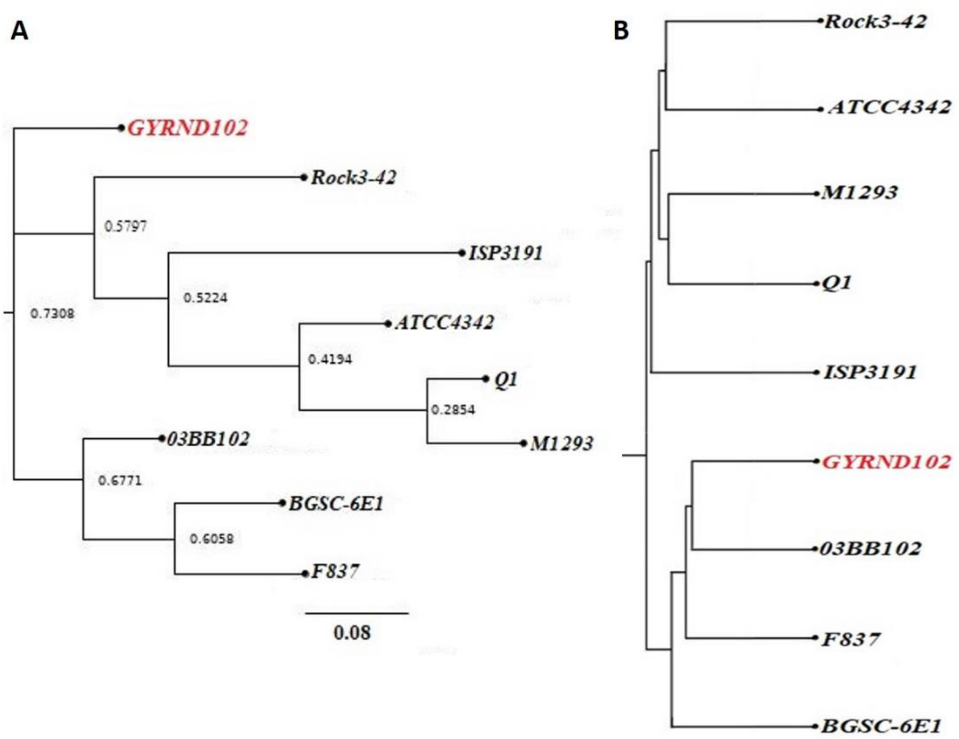

Figure S5. Phylogeny of the *B. cereus* strains based on the accessory genes analysis **(A)**, and core gene alignments **(B)**.

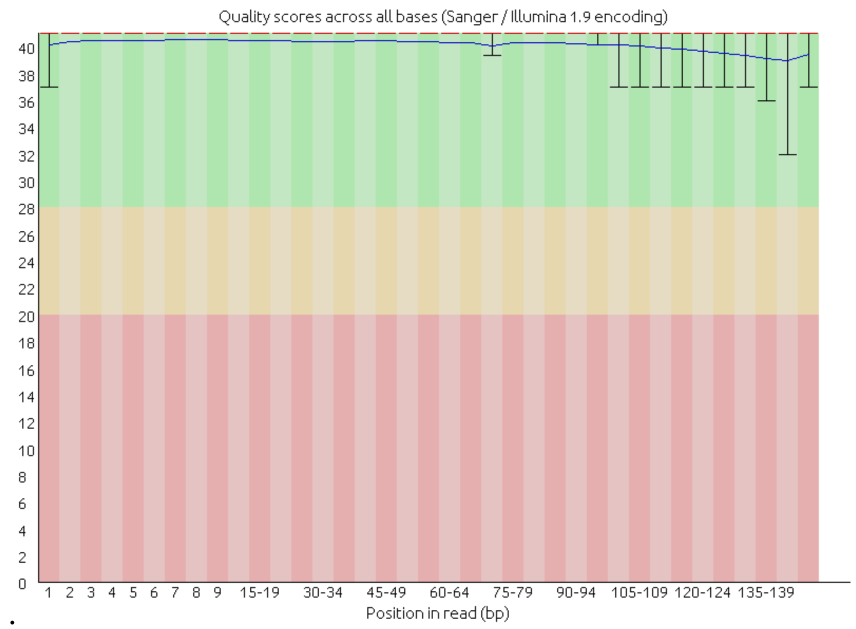

Figure S6. Quality scores across all bases after QC step

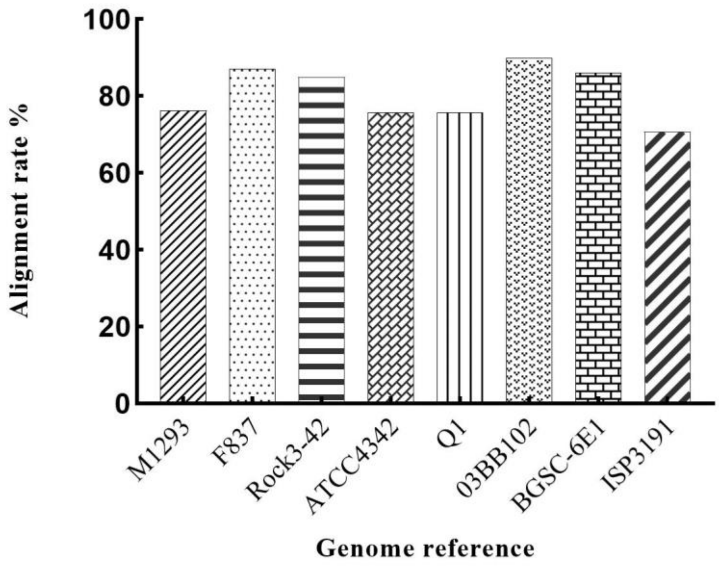

Figure S7. *B. cereus GYRND102* reads mapped to the 8 *B. cereus* strains

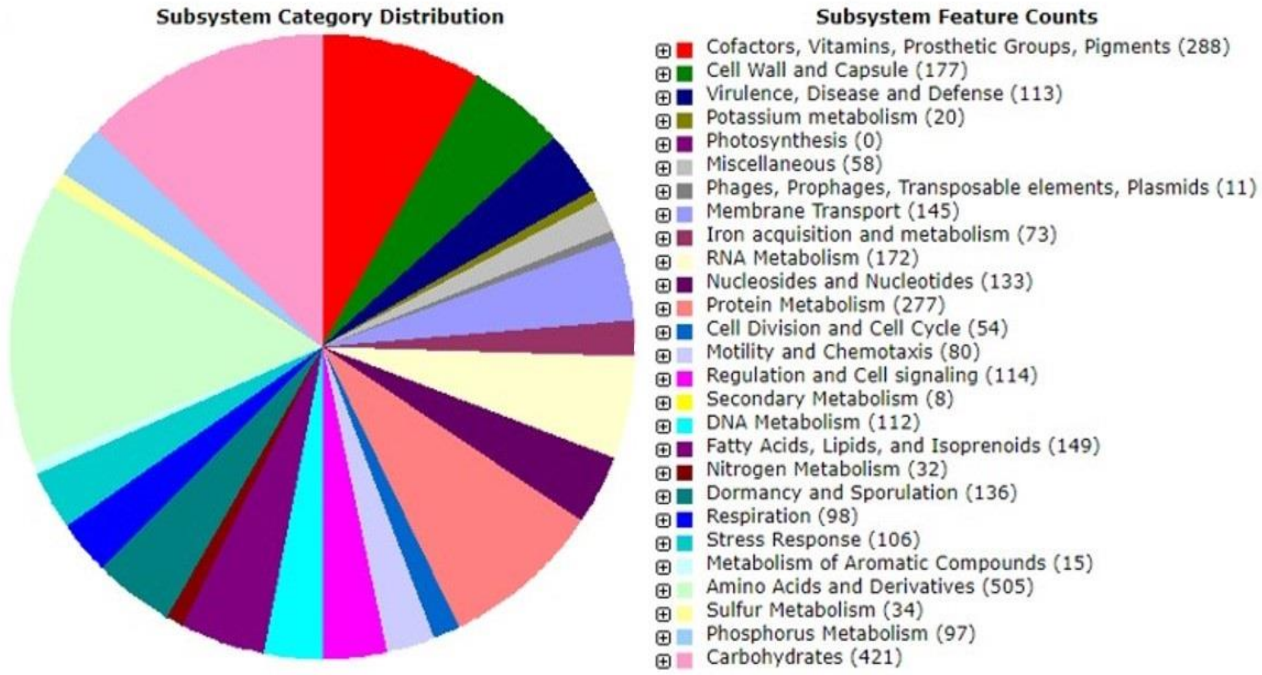

Figure S8. RAST subsystem annotation of *GYRND102* strain, the pie chart corresponds to the percentage of proteins commentated to each subsystem category. Subsystem categories are also listed clockwise in the legend from top to bottom.

Table S1 Study of the bacterial growth in presence of 10% toluene (v/v) and surveying the amount of toluene removal. The bacterial culture was performed in Mineral salt medium (NaCl 30 g/L) for 24 h.

Strain name	Strain source	OD in 600 nm	Toluene removal percentage
A	Isfahan Petrochemical (soil)	**0.2**	**6**
B	Isfahan Petrochemical (soil)	0.17	4.8
C	Isfahan Petrochemical (soil)	0.16	3.23
D	Isfahan Petrochemical (soil)	0.15	4.05
E	Isfahan Petrochemical (soil)	0.16	4.26
F	Morcheh Khort industrial site	0.1	2.26
G	Morcheh Khort industrial site	0.12	1.6
H	Morcheh Khort industrial site	0.1	0.5
I	Morcheh Khort industrial site	0.1	0.7
J	Morcheh Khort industrial site	0.13	3.4
K	Isfahan Petrochemical effluent	0.13	3.9
L	Isfahan Petrochemical effluent	0.12	1.4
M	lake (Meymeh County)	0.09	0.009
N	lake (Meymeh County)	0.08	0.05
O	lake (Meymeh County)	0.03	0.07
P	lake (Meymeh County)	0.05	0.09
Q	lake (Meymeh County)	0.09	0.04
R	Morcheh Khort industrial site	0.1	0.8

Table S2 Investigation of the mineral effect in reducing the COD and TOC amount as a result of *Bacillus cereus GYRND102* function in the petrochemical effluent

Parameter		Effluent containing no MSM	Effluent containing 0.905 g MSM	Effluent containing 1.810 g MSM
**COD**	**Initial**	90	90	90
	**24h**	84.2	81	74
	**48h**	77	74	70
**Efficiency (%)**		**15**	**18**	**22**
**TOC**	**Initial**	33	33	33
	**24h**	30.6	29	27
	**48h**	28.5	26	25
**Efficiency (%)**		**13.6**	**21**	**24**

Table S3 Investigation of the carbon source effect on increasing the COD and TOC reduction efficiency as a result of *Bacillus cereus GYRND102* activity in the effluent

Parameter		Effluent containing 0.5 g sucrose	Effluent containing 1 g sucrose	Effluent containing 2 g sucrose
COD	Initial	6105	11760	24300
	24h	5280	10050	21100
	48h	4100	6152	13900
Efficiency (%)		**32.5**	**47.6**	**42.7**
TOC	Initial	2010	3900	8190
	24h	1765	3340	7050
	48h	1400	2040	4650
Efficiency (%)		**30**	**47**	**43**

Table S4 Urban wastewater characteristics (entrance of Shahin Shahr wastewater treatment system in Isfahan)

Parameters	Urban wastewater
**pH**	7.15
**Turbidity**	184
**Electrical conductivity (EC)**	1146
**BOD**	386
**COD**	796
**TOC**	263

Table S5 Impact of municipal effluent and simultaneous use of municipal effluent and 0. 5 g sucrose on increasing the COD and TOC reduction efficiency as a result of *Bacillus cereus GYRND102* activity.

Parameter		Mixture of 50% petrochemical effluent and 50% municipal effluent	petrochemical effluent containing 0.5 g sucrose and 50% municipal effluent
COD	Initial	440	6200
	24h	391	5280
	48h	336	4290

**Efficiency (%)**		**24**	**30**
**TOC**	**Initial**	150	2030
	**24h**	132	1740
	**48h**	114	1445
**Efficiency (%)**		**24**	**28**

Table S6 The quality control of genomic DNA sample

Sample	Concentration (ng/µl)	λ 260 /λ 280	λ 260 /λ 230
**Genomic DNA**	2341.6	1.80	2.10

Table S7 Basic Statistics of the raw and clean sequences

Measure	Raw sequence	Clean sequence
**Reads (M)**	7,906,458	7,330,704
**Bases (b)**	1,185,968,700	1,062,952,082
**Read length (bp)**	150	145
**Q20(%)**	96.10	99.08
**GC(%)**	38	38

Note: Read (M): Total Number of Reads in million. Bases (b): Total Number of Bases in base. Q20: Base number percent summons with quality value of 20 or higher. GC%: The GC content percentage.

Table S8. The assembly quality assessment by QUAST

Tools	N50	max_length	Contig.no (>= 500 bp)	Total length (bp)
**SPAdes**	1,041,840	1,756,923	41	5,154,622

Table S9 BLAST search of 16*s rRNA* gene isolated from scaffold *GYRND102* in the NCBI GenBank database

Gene Marker	Database	Species Identified	Score (bits)	E-Value	Identification %
16_S rRNA	16s ribosomal RNA sequences (Bacteria and Archaea)	*Bacillus sp.* strain *P2*	1479	0.0	99. 40
		Bacillus sp. strain *KT-1*	1354	0.0	97.54
		Bacillus sp. strain *1NLA3E*	1343	0.0	95.73
		Bacillus sp. strain *S3*	1328	0.0	95.40

Table S10 Phylogenetic affiliation based on MLST analysis

Gene Markers	Databases	Identified Species	Query Cover (%)	E value	Per. Ident (%)
glpF	non-redundant protein sequences (nr)	*Bacillus thuringiensis str. Al Hakam*	100	0	100.100
		Bacillus cereus ATCC 14579	100	0	99.63
		Bacillus cereus	100	0	99.27
gmk		*Bacillus*	100	4e-146	100
		Bacillus cereus ATCC 10987	100	1e-145	100
		Bacillus cereus	100	2e-145	99.51
ilvD		*Bacillus cereus 03BB108*	100	0	100
		Bacillus cereus 03BB102	100	0	100
		Bacillus cereus group	100	0	99.64
pta		*Bacillus cereus*	100	0	100
		Bacillus cereus	100	0	99.38
		Bacillus cereus group	100	0	99.38
tpi		*Bacillus cereus AH1273*	100	0	99.60
		Bacillus cereus	100	0	99.60
		Bacillus cereus R309803	100	0	99.20
rpoB		*Bacillus cereus*	100	0	99.92
		Bacillus cereus	100	0	99.92
		Bacillus cereus	100	0	99.83
gyrB		*Bacillus cereus IS075*	100	0	99.84
		Bacillus sp. UMTAT18	100	0	99.69
		Bacillus cereus MSX-D12	100	0	99.53
mdh		*Bacillus cereus*	100	0	100
		Bacillus cereus	100	0	99.67
		Bacillus cereus	100	0	99.97
mbl		*Bacillus cereus FDAARGOS_797*	100	0	100
		Bacillus cereus D17	100	0	100
		Bacillus cereus FDAARGOS_802	100	0	99.88
muts		*Bacillus cereus*	100	0	99.89
		Bacillus cereus	100	0	99.89
		Bacillus cereus	100	0	99.89

Table S11 Reads mapped on the closest reference genome (*B. cereus* strain *03BB102*)

Sample	No. of reads	Total no. of bases	Percentage of reads passed filtering (%)	Longest read (bases)	Percentage of mapped read
***GYRND102*_clean_filtered_1.fq**	3,665,352	531,476,040	100.00	145	90.80
***GYRND102*_clean_filtered_2.fq**	3,665,352	531,476,040	100.00	145	90.70
**Total**	7,330,704	1,062,952,080	100.00	145	90.75

Table S12 Core- and Pan-genome analysis for *Bacillus cereus GYRND102* strain.

Trait	Genes	Annotation	Ko no.	*GYRND102*	*m1293*	*Rock3-42*	*Q1*	*ISP3191*	*F837*	*BGSC-6E1*	*ATCC4342*	*03BB102*
Oxidoreductases	*YdhR*	Putative monooxygenase YdhR	…	+	+	+	+	+	+	+	+	+
	hpaH	4-hydroxyphenylacetate 3-monooxygenase (Anthranilate 3-monooxygenase oxygenase component)	K00483	+	+	+			+	+	+	+
	pdhD_3	dihydrolipoyl dehydrogenase	K00382	+	+	+	+	+	+	+	+	+
	ACADM	Acyl-CoA dehydrogenase	K00249	+	+	+	+	+	+	+	+	+
	feaB (styD_1)	phenylacetaldehyde dehydrogenase	K00146	+	+	+	+	+	+	+	+	+
	…	Zinc-type alcohol dehydrogenase-like protein	K00344	+								
	dehH10	Haloacetate dehalogenase H-1	K01561	+								
Transcription factors	*yybR*	putative HTH-type transcriptional regulator YybR	…	+	+	+	+	+	+	+	+	+
	gmuR	HTH-type transcriptional regulator GmuR	K03492	+	+	+	+	+	+	+	+	+
	yxaF	putative HTH-type transcriptional regulator YxaF	K18939	+	+	+	+	+	+	+	+	+
	gltR_3	HTH-type transcriptional regulator GltR	K23773	+	+	+	+	+	+	+	+	+
	gltC_1	HTH-type transcriptional regulator GltC	K19338	+	+	+	+	+	+	+	+	+
	lytR_2	Transcriptional regulator LytR	K01005	+	+	+	+	+	+	+	+	+
	yofA_1	HTH-type transcriptional regulator YofA	K23773	+	+	+	+	+	+	+	+	+
	lutR_2	HTH-type transcriptional regulator LutR	K05799	+	+	+	+	+	+	+	+	+
	ttgR	HTH-type transcriptional regulator TtgR	…									
	cynR	HTH-type transcriptional regulator CynR	K11921	+	+	+	+	+	+	+	+	+
	hxlR	*HTH-type transcriptional activator HxlR*	…	+								
	mhqR_4	*HTH-type transcriptional regulator MhqR*	K15973	+								
Two-component system	*phoR*	OmpR family, phosphate regulon sensor histidine kinase	K07636	+	+	+	+	+	+	+	+	+
	phoP (phoB)	OmpR family, alkaline phosphatase synthesis response regulator	K07658	+	+	+	+	+	+	+	+	+
	phoA (phoB)	alkaline phosphatase	K01077	+	+	+	+	+	+	+	+	+
	liaS	NarL family, sensor histidine kinase LiaS	K11617	+	+	+	+	+	+	+	+	+
	liaR	NarL family, response regulator LiaR	K11618	+	+	+	+	+	+	+	+	+
	liaI	lia operon protein LiaI	K11619	+								
	liaF	lia operon protein LiaF	K11622	+								
	yufL (malK)	CitB family, sensor histidine kinase MalK	K11614	+		+	+	+	+	+	+	+
	malR	CitB family, response regulator MalR	K11615	+		+	+	+	+	+	+	+
	maeA (mdh)	malate dehydrogenase	K00027	+	+	+	+	+	+	+	+	+
	maeN	malate:Na+ symporter/L-malate permease	K11616	+		+	+	+	+	+	+	+
	glnK	sensor histidine kinase GlnK	K07717	+	+	+	+	+	+	+	+	+
	glsA	glutaminase	K01425	+	+	+	+	+	+	+	+	+
	glnL	response regulator GlnL	K07719	+	+	+	+	+	+	+	+	+
Sporulation family	*kinB*	sporulation sensor kinase B	K07697	+								
	kapB	kinase-associated protein B	K06347	+	+	+	+		+	+	+	+
	kinD	sporulation sensor kinase D	K13532	+	+	+	+	+	+	+	+	+
	kinE	Sporulation kinase E	K13533	+	+	+	+	+	+	+	+	+
	spo0F	stage 0 sporulation protein F (response regulator)	K02490	+	+	+	+		+	+	+	+
	spo0B	stage 0 sporulation protein B (sporulation initiation phosphotransferase)	K06375	+	+	+	+	+	+	+	+	+
	spo0A	stage 0 sporulation protein A (response regulator)	K07699	+	+	+	+	+	+	+	+	+
	spo0M	Sporulation-control protein	K06377	+	+	+	+	+	+	+	+	+
…	*qorA*	Quinone oxidoreductase 1/ NADH:ubiquinone oxidoreductase complex assembly factor 3	K09008	+	+	+	+	+	+	+	+	+
	YhfP	Putative quinone oxidoreductase	K19745	+	+	+	+	+	+	+	+	+
	COQ3 (ubiG_1)	Ubiquinone biosynthesis O-methyltransferase	K00591	+	+	+	+	+	+	+	+	+
	menG_4	Demethylmenaquinone methyltransferase	K03183	+	+	+	+	+	+	+	+	+

	mqo1	putative malate:quinone oxidoreductase 1	K00116	+	+	+	+	+	+	+	+	+
Transporters	*znuB*	High-affinity zinc uptake system membrane protein ZnuB	K09816	+	+	+	+	+	+	+	+	+
	glpF	Glycerol uptake facilitator protein	K02440	+	+	+	+	+	+	+	+	+
	queT	Magnesium and cobalt efflux protein CorC	K16302	+	+	+	+	+	+	+	+	+
	panS	Pantothenate precursors transporter PanS	K03453	+	+	+	+	+	+	+	+	+
Lyases	*mdeA*	L-methionine gamma-lyase (O-succinylhomoserine sulfhydrylase)	K01761	+	+	+	+	+	+	+	+	+
	metI	Cystathionine gamma-synthase/O-acetylhomoserine (thiol)-lyase	K01740	+	+	+	+	+	+	+	+	+
...	*pinR*	Putative DNA-invertase from lambdoid prophage Rac	K14060	+	+	+	+	+	+	+	+	+
Hydrolases	*pcrA*	ATP-dependent DNA helicase UvrD1	K03657	+	+	+	+	+	+	+	+	+
	recG	ATP-dependent DNA helicase RecG	K03655	+	+	+	+	+	+	+	+	+
	ycfH	putative metal-dependent hydrolase YcfH	K03424	+	+	+	+	+	+	+	+	+
	suhB_2	Inositol-1-monophosphatase/ myo-inositol-1(or 4)-monophosphatase	K01092	+								
	suhB_3	Inositol-1-monophosphatase	K01092	+								
	NUDT2	Bis(5'-nucleosyl)-tetraphosphatase	K01518	+								
Methyltransferases	*hpaIIM*	DNA (cytosine-5)-methyltransferase 1/ Modification methylase HpaII	K00558							+		+
	hpnR	hopanoid C-3 methylase	K22704						+			
ABC transporters	*bioY2*	Biotin transporter BioY2	K03523	+	+	+	+		+	+	+	+
	ytrB_2	ABC transporter ATP-binding protein YtrB	K01990	+	+	+	+	+	+	+		+
	ybiT_4	putative ABC transporter ATP-binding protein YbiT	…	+	+	+	+		+	+	+	+
Toxin-antitoxin system	*hipB_2*	antitoxin/DNA-binding transcriptional repressor HipB	K15773		+		+					+
	yxxD	Antitoxin YxxD	K21494	+					+	+		+
	ndoAI	Antitoxin EndoAI	K07723	+	+	+	+	+	+	+	+	+
	higA1	Antitoxin HigA1	K18831	+	+	+	+	+	+	+	+	+
Phosphotransferases	*glpK*	Glycerol kinase	K00864	+	+	+	+	+	+	+	+	+
	coaE_1	Dephospho-CoA kinase	K00859	+	+	+	+	+		+	+	+
	pdxK	Pyridoxine kinase	K00868	+	+	+	+		+	+	+	+
Acetyl transferase	*ttr*	N-alpha-acetyltransferase	K20791	+	+	+	+	+	+	+	+	+
Alkyl transferase	*aroF*	Phospho-2-dehydro-3-deoxyheptonate aldolase, Tyr-sensitive	K01626	+	+	+	+	+	+	+	+	+
	trmB	tRNA (guanine-N(7)-)-methyltransferase / N7-methylguanine methylase	K03439	+	+	+	+	+	+	+	+	+
	pcm	Protein-L-isoaspartate O-methyltransferase	K00573	+	+	+	+	+	+	+	+	+
Putative transposase	*Int-Tn_2*	Transposase from transposon Tn916	…	+					+	+	+	+
	...	putative transposase	K07497	+								
	…	putative transposase	K07496	+								

Antibiotic resistance	*penP*	beta-lactamase class A	K17836	+	+	+	+	+	+	+	+	+
	bla2 (blaB)	metallo-beta-lactamase class B	K17837	+	+	+	+	+	+	+	+	+
	mrcA	penicillin-binding protein 1A	K05366	+	+	+	+	+	+	+	+	+
	pbp2A	penicillin-binding protein 2A	K12555	+	+	+	+	+	+	+	+	+
	catA	chloramphenicol acetyltransferase	K19271	+	+	+	+	+	+	+	+	+
	vat	virginiamycin A acetyltransferase	K18234	+	+	+	+	+		+	+	+
	ereA_B	erythromycin esterase	K06880	+								
	vanW	Vancomycin B-type resistance protein VanW	K18346	+	+	+	+	+	+	+	+	+
	tetM (tetO)	ribosomal protection tetracycline resistance protein	K18220	+		+			+		+	+
	amiA	N-acetylmuramoyl-L-alanine amidase	K01448	+	+	+	+	+	+	+	+	+
	dltC	D-alanine--poly(phosphoribitol) ligase subunit 2	K14188	+	+	+	+	+	+	+	+	+
	dltA	D-alanine--poly(phosphoribitol) ligase subunit 1	K03367	+	+	+	+	+	+	+	+	+
	mprF	phosphatidylglycerol lysyltransferase	K14205	+	+	+	+	+	+	+	+	+
	aacC	aminoglycoside 3-N-acetyltransferase	K00662	+								
	aadK	aminoglycoside 6-adenylyltransferase	K05593	+	+	+	+	+	+	+	+	+
	aacA	Aminoglycoside N(6')-acetyltransferase	K00663	+	+	+	+	+	+	+	+	+
…	*YbbJ*	Inner membrane protein YbbJ	K07340	+	+	+	+	+	+	+	+	+
	YohD	Inner membrane protein YohD	…	+	+	+	+	+	+	+	+	+
	YbiR	Inner membrane protein YbiR	…				+	+	+	+	+	
	YohK	Inner membrane protein YohK	…	+	+	+	+	+	+	+	+	+
	YgaZ	Inner membrane protein YgaZ	…	+	+	+	+	+	+	+	+	+
	alx	Inner membrane protein alx	…	+	+	+	+	+	+	+	+	+
	YiaA	Inner membrane protein YiaA	…	+	+	+	+	+	+	+	+	+
	YabI	Inner membrane protein YabI	…	+	+	+	+	+	+	+	+	+
DNA replication protein	*dnaI*	Primosomal protein DnaI	K02317	+	+	+	+	+	+	+	+	+
	polA	DNA polymerase I	K02335	+	+	+	+	+	+	+	+	+
Ortho-cleavage pathway	*catA*	catechol 1,2-dioxygenase	K03381									
	catB	muconate cycloisomerase	K01856									
	catC	muconolactone D-isomerase	K03464									
	pcaD	3-oxoadipate enol-lactonase	K01055									
	pcaL	4-carboxymuconolactone decarboxylase	K14727									
Meta-cleavage pathway	*catE*	catechol 2,3-dioxygenase	K07104	+	+	+	+	+	+	+	+	+
	dmpC (xylG)	Aminomuconate-semialdehyde /2-hydroxymuconate-6-semialdehyde dehydrogenase	K10217	+								
	dmpI (xylH)	4-oxalocrotonate tautomerase	K01821	+	+	+	+	+	+	+	+	+
	xylI	2-oxo-3-hexenedioate decarboxylase	K01617	+								
	dmpD (xylF)	2-hydroxymuconate-semialdehyde hydrolase	K10216	+	+	+	+	+	+	+	+	+
	mhpD (xylJ)	2-keto-4-pentenoate hydratase	K02554 (K18364)	+	+	+			+	+	+	+
	mhpE (dmpG)	4-hydroxy 2-oxovalerate aldolase	K01666	+	+	+	+	+	+	+	+	+
	mhpF	acetaldehyde dehydrogenase	K04073	+	+	+			+	+	+	+
CRISPR-Cas syst	*cas9 (csn1)*	CRISPR-associated endonuclease Csn1	K09952				+					
	cas2 (ygbF)	CRISPR-associated protein Cas2	K09951	+	+	+	+	+	+	+	+	
…	***hpxO_2***	FAD-dependent urate hydroxylase	…	+								

Table S13 Gene annotation of *Bacillus cereus GYRND102* strain genome based on genes implicated in meta-cleavage pathway

Traits	KEGG Gene Name	Ko number	Activity	EC number
**Catechol meta-**	*catE*	K07104	catechol 2,3-dioxygenase	1.13.11.2
	dmpC	K10217	Aminomuconate-semialdehyde	1.2.1.32

	praB (*xylG*)		2-hydroxymuconate-6-semialdehyde dehydrogenase	1.2.1.85
	dmpI (*xylH*)	K01821	4-oxalocrotonate tautomerase / 4-oxalocrotonate isomerase	5.3.2.6
	dmpH (*xylI*)	K01617	2-oxo-3-hexenedioate decarboxylase	4.1.1.77
	dmpD (*xylF*)	K10216	2-hydroxymuconate-semialdehyde hydrolase	3.7.1.9
	dmpH (*mhpD*)	K02554	2-keto-4-pentenoate hydratase	4.2.1.80
	dmpG (*mhpE*)	K01666	4-hydroxy 2-oxovalerate aldolase	4.1.3.39
	mhpF	K04073	acetaldehyde dehydrogenase	1.2.1.10

Table S14 Genes contributing in aromatic compounds degradation pathways in *Bacillus cereus GYRND102* strain genome based on gene annotation.

KEGG Pathway		Ko number	EC number	KEGG Gene Name	Definition
Benzoate degradation	Xenobiotics biodegradation and metabolism	K00074	EC:1.1.1.157	*paaH*	3-hydroxybutyryl-CoA dehydrogenase
		K00626	EC:2.3.1.9	*ACAT*	acetyl-CoA C-acetyltransferase
		K01607	EC:4.1.1.44	*pcaC*	4-carboxymuconolactone decarboxylase
		K01617	EC:4.1.1.77	*dmpH* (*xylI*)	2-oxo-3-hexenedioate decarboxylase
		K01666	EC:4.1.3.39	mhpE	4-hydroxy 2-oxovalerate aldolase
		K01821	EC:5.3.2.6	*praC (xylH)*	4-oxalocrotonate tautomerase
		K02554	EC:4.2.1.80	*mhpD*	2-keto-4-pentenoate hydratase
		K04073	EC:1.2.1.10	*mhpF*	acetaldehyde dehydrogenase
		K07104	EC:1.13.11.2	*catE*	catechol 2,3-dioxygenase
		K10217	EC:1.2.1.85	*praB* (*xylG*)	2-hydroxymuconate-6-semialdehyde dehydrogenase
			EC:1.2.1.32	*dmpC*	aminomuconate-semialdehyde
		K13767	EC:4.2.1.17	*fadB*	enoyl-CoA hydratase
		K00632	EC:2.3.1.16	*fadA (fadI)*	acetyl-CoA acyltransferase
		K07516	EC:1.1.1.35	*fadN*	3-hydroxyacyl-CoA dehydrogenase
		K00141	EC:1.2.1.28	*xylC*	benzaldehyde dehydrogenase
Aminobenzoate degradation		K01034	EC:2.8.3.8<br>EC: 2.8.3.9	*atoD*	acetate CoA<br>acetoacetate CoA-transferase alpha subunit
		K01035	EC:2.8.3.8<br>EC: 2.8.3.9	*atoA*	acetate CoA<br>acetoacetate CoA-transferase beta subunit
		K01426	EC:3.5.1.4	*amiE*	amidase
		K14338	EC:1.14.14.1<br>EC:1.6.2.4	*cypD_E(CYP102A, CYP505)*	cytochrome P450<br>NADPH-cytochrome P450 reductase
		K01101	EC:3.1.3.41		4-nitrophenyl phosphatase
Chloroalkane and chloroalkene degradation		K00121	EC: 1.1.1.284 / EC:1.1.1.1	*frmA (ADH5)*	S-(hydroxymethyl)glutathione dehydrogenase / alcohol dehydrogenase
		K00128	EC:1.2.1.3	*ALDH*	aldehyde dehydrogenase (NAD+)
		K01560	EC:3.8.1.2	*ncr*	2-haloacid dehalogenase
		K04072	EC:1.2.1.10<br>EC:1.1.1.1	*adhE*	acetaldehyde dehydrogenase<br>alcohol dehydrogenase
		K13953	EC: 1.1.1.1	*adhP*	alcohol dehydrogenase, propanol-preferring
		K00001	EC: 1.1.1.1	*adh*	alcohol dehydrogenase
		K07104	EC:1.13.11.2	*catE*	catechol 2,3-dioxygenase
		K10680	EC:1.-.-.-	*nemA*	N-ethylmaleimide reductase
Xylene degradation		K01617	EC:4.1.1.77	*dmpH (xylI)*	2-oxo-3-hexenedioate decarboxylase
		K01666	EC:4.1.3.39	*mhpE*	4-hydroxy 2-oxovalerate aldolase
		K01821	EC:5.3.2.6	*praC (xylH)*	4-oxalocrotonate tautomerase
		K02554	EC:4.2.1.80	*mhpD*	2-keto-4-pentenoate hydratase
		K04073	EC:1.2.1.10	*mhpF*	acetaldehyde dehydrogenase
		K07104	EC:1.13.11.2	*catE*	catechol 2,3-dioxygenase
		K10217	EC:1.2.1.85	*praB (xylG)*	2-hydroxymuconate-6-semialdehyde dehydrogenase
			EC:1.2.1.32	*dmpC*	aminomuconate-semialdehyde
Styrene		K01426	EC:3.5.1.4	*amiE*	amidase
		K07104	EC:1.13.11.2	*catE*	catechol 2,3-dioxygenase

	K00451	EC:1.13.11.5	*hmgA(HGD)*	homogentisate 1,2-dioxygenase
Dioxin degradation	K01617	EC:4.1.1.77	*dmpH (xylI)*	2-oxo-3-hexenedioate decarboxylase
	K01666	EC:4.1.3.39	*mhpE*	4-hydroxy 2-oxovalerate aldolase
	K01821	EC:5.3.2.6	*praC (xylH)*	4-oxalocrotonate tautomerase
	K02554	EC:4.2.1.80	*mhpD*	2-keto-4-pentenoate hydratase
	K04073	EC:1.2.1.10	*mhpF*	acetaldehyde dehydrogenase
Naphthalene degradation	K00001	EC:1.1.1.1	*adh*	alcohol dehydrogenase
	K00121	EC: 1.1.1.284 / EC:1.1.1.1	*frmA (ADH5)*	S-(hydroxymethyl)glutathione dehydrogenase / alcohol dehydrogenase
	K04072	EC:1.2.1.10 EC:1.1.1.1	*adhE*	acetaldehyde dehydrogenase alcohol dehydrogenase
	K13953	EC 1.1.1.1	*adhP*	alcohol dehydrogenase, propanol-preferring
Metabolism of xenobiotics by cytochrome p450	K00121	EC: 1.1.1.284 / EC:1.1.1.1	*frmA (ADH5)*	S-(hydroxymethyl)glutathione dehydrogenase / alcohol dehydrogenase
	K00001	EC:1.1.1.1	*adh*	alcohol dehydrogenase
	K13953	EC 1.1.1.1	*adhP*	alcohol dehydrogenase, propanol-preferring
Flavin-dependent monooxygenase	…	EC: 1.14.13.-	...	Nitrilotriacetate monooxygenase component B (flavin-dependent monooxygenase)
	K04091	EC: 1.14.14.5	*ssuD*	Alkanesulfonate monooxygenase
	K16901	EC: 1.14.14.8	*bca*	anthranilate 3-monooxygenase (FAD) 4-hydroxyphenylacetate 3-monooxygenase
		EC: 1.14.14.9	*HpaB*	
	K15760	EC: **1.14.13.-**	*tmoA*	Toluene-4-monooxygenase system protein A
	K00494	EC: 1.14.14.3	*luxA*	Alkanal monooxygenase alpha chain
	K00490	EC: 1.14.14.1	*CYP4F*	Flavocytochrome P450/ Xenobiotic monooxygenase/ Flavoprotein-linked monooxygenase
	K10215	EC: 1.14.13.-	*EthA*	monooxygenase
...	K00219	EC 1.3.1.34	*fadH*	2,4-dienoyl-CoA reductase (NADPH2)

Table S15 Genes related to secretion systems and flagellar assembly in *Bacillus cereus GYRND102* strain genome according to gene annotation

**KEGG Pathway**	**Type**	**Ko number**	**EC number**	**KEGG Gene Name**	**Definition**
Flagellar assembly	Basal body Hook	K02408	…	*fliE*	flagellar hook-basal body complex protein FliE
		K02409	…	*fliF*	flagellar M-ring protein FliF
		K02410	…	*fliG*	flagellar motor switch protein FliG
		K02411	…	*fliH*	flagellar assembly protein FliH
		K02412	EC:7.4.2.8	*fliI*	flagellum-specific ATP synthase
		K02416	…	*fliM*	flagellar motor switch protein FliM
		K02417	…	*fliN*	flagellar motor switch protein FliN
		K02419	…	*fliP*	flagellar biosynthesis protein FliP
		K02420	…	*fliQ*	flagellar biosynthesis protein FliQ
		K02421	…	*fliR*	flagellar biosynthesis protein FliR
		K02387	…	*flgB*	flagellar basal-body rod protein FlgB
		K02388	…	*flgC*	flagellar basal-body rod protein FlgC
		K02389	…	*flgD*	flagellar basal-body rod modification protein FlgD

		K02390	…	*flgE*	flagellar hook protein FlgE
		K02392	…	*flgG*	flagellar basal-body rod protein FlgG
		K02396	…	*flgK*	flagellar hook-associated protein 1
		K02397	…	*flgL*	flagellar hook-associated protein 3 FlgL
	T3SS	K02400	…	*flhA*	flagellar biosynthesis protein FlhA
		K02401	…	*flhB*	flagellar biosynthesis protein FlhB
	Filament	K02406	…	*fliC(hag)*	flagellin
		K02407	…	*fliD*	flagellar hook-associated protein 2
		K02422	…	*fliS*	flagellar secretion chaperone FliS
Bacterial secretion system	Sec-SRP	K03070	...	*secA*	preprotein translocase subunit SecA(ATPase)
		K03072	...	*secD*	preprotein translocase subunit SecD
		K03073	...	*secE*	preprotein translocase subunit SecE
		K03074	...	*secF*	preprotein translocase subunit SecF
		K03075	…	*secG*	preprotein translocase subunit SecG
		K03076	...	*secY*	preprotein translocase subunit SecY
		K03106	EC: 3.6.5.4	*ffh*	signal recognition particle subunit SRP54
		K03110	...	*ftsY*	fused signal recognition particle receptor
		K03210	...	*yajC*	preprotein translocase subunit YajC
		K03217	…	*YidC*	YidC/Oxa1 family membrane protein insertase
	Tat	K03116	…	*tatA*	sec-independent protein translocase protein TatA
		K03117	...	*tatB*	sec-independent protein translocase protein TatB
		K03118	...	*tatC*	sec-independent protein translocase protein TatC

Table S16 Genes involved in antibiotic resistance pathways according to gene annotation of *Bacillus* *cereus* *GYRND102* strain genome

KEGG Pathway	Ko number	EC number	KEGG Gene Name	Definition
beta-Lactam resistance	K02171	…	*blaI*	BlaI family transcriptional regulator (penicillinase repressor)
	K02172	…	*blaR1*	bla regulator protein blaR1
	K10823	…	*oppF*	oligopeptide transport system ATP-binding protein
	K05366	EC 2.4.1.129 / 3.4.16.4	*mrcA*	penicillin-binding protein 1A
	K12555	EC:2.4.1.129/ 3.4.16.4	*pbp2A*	penicillin-binding protein 2A
	K15580	…	*oppA(mppA)*	oligopeptide transport system substrate-binding protein
	K15581	…	*oppB*	oligopeptide transport system permease protein
	K15582	…	*oppC*	oligopeptide transport system permease protein
	K15583	…	*oppD*	oligopeptide transport system ATP-binding protein
	K17836	EC3.5.2.6	penP	beta-lactamase class B
	K17837	EC3.5.2.6	*bla2(blaB)*	Metallo-beta-lactamase class B
	K18104	EC7.6.2.2	*abcA(bmrA)*	ATP-binding cassette, subfamily B, bacterial AbcA/BmrA
Vancomycin resistance	K01000	EC 2.7.8.13	*mraY*	phospho-N-acetylmuramoyl-pentapeptide-transferase
	K01775	EC 5.1.1.1	*alr*	alanine racemase
	K01921	EC 6.3.2.4	*ddl*	D-alanine-D-alanine ligase
	K01929	EC 6.3.2.10	*murF*	UDP-N-acetylmuramoyl-tripeptide--D-alanyl-D-alanine ligase
	K02563	EC 2.4.1.227	*murG*	UDP-N-acetylglucosamine--N-acetylmuramyl-(pentapeptide)
	K07260	EC:3.4.17.14	*vanY*	zinc D-Ala-D-Ala carboxypeptidase

	K18350	EC:2.7.13.3	*vanSC(vanSE)*	OmpR family, sensor histidine kinase VanS
	K18344	…	*vanRB(vanR)*	OmpR family, response regulator VanR
Cationic antimicrobial peptide	K01448	EC 3.5.1.28	*amiA*	N-acetylmuramoyl-L-alanine amidase
	K03367	EC 6.1.1.13	*dltA*	D-alanine--poly(phosphoribitol) ligase subunit 1
	K03739	…	*dltB*	membrane protein involved in D-alanine export
	K03740	…	*dltD*	D-alanine transfer protein
	K14188	EC 6.1.1.13	*dltC*	D-alanine--poly(phosphoribitol) ligase subunit 2
	K04771	EC 3.4.21.107	*degP*	serine protease Do
	K18346	…	*vanW*	Vancomycin B-type resistance protein
	K14205	EC 2.3.2.3	*mprF*	phosphatidylglycerol lysyltransferase
Antimicrobial resistance genes	K00662	EC:2.3.1.81	*aacC*	aminoglycoside 3-N-acetyltransferase
	K00663	EC:2.3.1.82	*aacA*	aminoglycoside 6'-N-acetyltransferase
	K05593	EC:2.7.7.-	*aadK*	aminoglycoside 6-adenylyltransferase
	K06880	EC:3.1.1.-	*ereA_B*	erythromycin esterase
	K06979	…	*mph*	macrolide phosphotransferase
	K08168	…	*tetB*	MFS transporter, DHA2 family, metal-tetracycline-proton antiporter
	K08217	…	*mef*	MFS transporter, DHA3 family, macrolide efflux protein
	K17910	EC:2.7.1.190	*aphD*	aminoglycoside 2''-phosphotransferase
	K18220	…	*tetM(tetO)*	ribosomal protection tetracycline resistance protein
	K18555	…	*qnr(mcbG)*	fluoroquinolone resistance protein
	K18907	…	*norG*	GntR family transcriptional regulator (regulator for abcA and norABC)
	K19271	EC:2.3.1.28	*catA*	chloramphenicol O-acetyltransferase type A
	K18231	…	*msr(vmlR)*	macrolide transport system ATP-binding/permease protein
	K18234	EC:2.3.1.-	*vat*	virginiamycin A acetyltransferase

Table S17 Genes associated with Quorum sensing process based on gene annotation of *Bacillus* cereus *GYRND102* strain genome

KEGG Pathway	Ko number	EC number	KEGG Gene Name	Definition
Quorum sensing system	K08321	EC:2.3.1.245	*lsrF*	3-hydroxy-5-phosphonooxypentane-2,4-dione thiolase
	K10557	…	*lsrD*	AI-2 transport system permease protein
	K01657	EC:4.1.3.27	*trpE*	anthranilate synthase component I (phnA)
	K01658	EC:4.1.3.27	*trpG*	anthranilate synthase component II (phnB)
	K01897	EC:6.2.1.3	*ACSL*	long-chain acyl-CoA synthetase
	K01995	...	*livG*	branched-chain amino acid transport system ATP-binding protein
	K01996	...	*livF*	branched-chain amino acid transport system ATP-binding protein
	K01997	...	*livH*	branched-chain amino acid transport system permease protein
	K01998	...	*livM*	branched-chain amino acid transport system permease protein
	K01999	...	*livK*	branched-chain amino acid transport system substrate-binding protein
	K10556	…	*lsrC*	AI-2 transport system permease protein
	K11531	…	*lsrR*	lsr operon transcriptional repressor
	K10555	…	*lsrB*	AI-2 transport system substrate-binding protein
	K11216	EC:2.7.1.189	*lsrK*	autoinducer-2 kinase
	K03070	...	*secA*	preprotein translocase subunit SecA

K10558	…	*lsrA(ego)*	AI-2 transport system ATP-binding protein
K03073	...	*secE*	preprotein translocase subunit SecE
K03076	...	*secY*	preprotein translocase subunit SecY
K07173	EC:4.4.1.21	*luxS*	S-ribosylhomocysteine lyase
K13075	EC:3.1.1.81	*ahlD(aiiA)*	N-acyl homoserine lactone hydrolase
K03106	EC:3.6.5.4	*SRP54*	signal recognition particle subunit SRP54
K03110	...	*ftsY*	fused signal recognition particle receptor
K03210	...	*yajC*	preprotein translocase subunit YajC
K03217	...	*yidC*	YidC/Oxa1 family membrane protein insertase
K03666	...	*hfq*	host factor-I protein
K07706	EC:2.7.13.3	*agrC(blpH)*	LytTR family, sensor histidine kinase AgrC
K07707	…	*agrA(blpR)*	LytTR family, response regulator AgrA
K11031	…	*slo*	thiol-activated cytolysin
K09936	...	*TC.BAT2*	bacterial/archaeal transporter family-2 protein (ToxF)
K14983	…	*ciaR*	OmpR family, response regulator CiaR
K15580	…	*oppA(mppA)*	oligopeptide transport system substrate-binding protein
K11752	EC 3.5.4.26 / 1.1.1.193	*ribD*	diaminohydroxyphosphoribosylaminopyrimidine deaminase / 5-amino-6-(5-phosphoribosylamino) uracil reductase (ToxE)
K02035	…	*ABC.PE.S*	peptide/nickel transport system substrate-binding protein
K02250	…	*comK*	competence protein ComK
K06361	EC:3.1.-. -	*rapC*	response regulator aspartate phosphatase
K12257	…	*secDF*	SecD/SecF fusion protein
K02490	…	*spo0F*	stage 0 sporulation protein F (response regulator)
K06375	EC:2.7.-. -	*spo0B*	stage 0 sporulation protein B (sporulation initiation phosphotransferase)
K20390	…	*papR*	regulatory peptide PapR
K08777	EC:3.4.24.-	*nprB*	neutral peptidase B
K20481	…	*nprX(nprRB)*	regulatory peptide NprX
K20391		*plcR*	HTH-type transcriptional regulator, pleiotropic regulator of extracellular virulence genes
K20480	…	*nprR(nprA)*	HTH-type transcriptional regulator, quorum sensing regulator NprR
K01114	EC:3.1.4.3	*plc*	phospholipase C
K11033	…	*nheA*	non-hemolytic enterotoxin A
K02031	…	*ddpD*	peptide/nickel transport system ATP-binding protein
K02032	…	*ddpF*	peptide/nickel transport system ATP-binding protein
K02033	…	*ABC.PE.P*	peptide/nickel transport system permease protein
K02034	…	*ABC.PE.P1*	peptide/nickel transport system permease protein
K02035	…	*ABC.PE.S*	peptide/nickel transport system substrate-binding protein
K07699	…	*spo0A*	response regulator, stage 0 sporulation protein A
K10823	…	*oppF*	oligopeptide transport system ATP-binding protein
K11034	…	*nheBC*	non-hemolytic enterotoxin B/C
K15581	…	*oppB*	oligopeptide transport system permease protein
K15582	…	*oppC*	oligopeptide transport system permease protein
K15583	…	*oppD*	oligopeptide transport system ATP-binding protein
K02031	…	*ddpD*	peptide/nickel transport system ATP-binding protein
K02032	…	*ddpF*	peptide/nickel transport system ATP-binding protein
K02033	…	*ABC.PE.P*	peptide/nickel transport system permease protein
K02034	…	*ABC.PE.P1*	peptide/nickel transport system permease protein

Table S18 Gene annotation of *Bacillus cereus GYRND102* strain genome based on genes contributed in two-component regulatory system

KEGG Pathway	Ko number	EC number	KEGG Gene Name	Definition
**Two-component system**	K07658	…	*phoP(phoB1)*	OmpR family, alkaline phosphatase synthesis response regulator PhoP
	K01077	EC:3.1.3.1	*phoA(phoB)*	alkaline phosphatase
	K00370	EC:1.7.5.1/ 1.7.99.-	*narG(narZ)*	nitrate reductase / nitrite oxidoreductase, alpha subunit
	K00371	EC:1.7.5.1/ 1.7.99.-	*narH(narY)*	nitrate reductase / nitrite oxidoreductase, beta subunit
	K00374	EC:1.7.5.1/ 1.7.99.-	*narI(narV)*	nitrate reductase gamma subunit
	K00373	...	*narJ(narW)*	nitrate reductase molybdenum cofactor assembly chaperone NarJ/NarW
	K14988	...	*salK*	NarL family, secretion system sensor histidine kinase SalK
	K00425	EC 1.10.3.14	*cydA*	cytochrome d ubiquinol oxidase subunit I
	K00426	EC 1.10.3.14	*cydB*	cytochrome d ubiquinol oxidase subunit II
	K00626	EC 2.3.1.9	*atoB*	acetyl-CoA C-acetyltransferase
	K14989	...	*salR*	NarL family, secretion system response regulator SalR
	K01915	EC 6.3.1.2	*glnA*	glutamine synthetase
	K02040	...	*pstS*	phosphate transport system substrate-binding protein
	K02106	...	*atoE*	short-chain fatty acids transporter
	K02259	...	*COX15*	cytochrome c oxidase assembly protein subunit 15
	K02313	...	*dnaA*	chromosomal replication initiator protein
	K07646	EC:2.7.13.3	*kdpD*	OmpR family, sensor histidine kinase KdpD
	K01546	...	*kdpA*	potassium-transporting ATPase potassium-binding subunit
	K02406	...	*fliC*	flagellin
	K01547	EC:7.2.2.6	*kdpB*	potassium-transporting ATPase ATP-binding subunit
	K02556	...	*motA*	chemotaxis protein MotA
	K01548	...	*kdpC*	potassium-transporting ATPase KdpC subunit
	K07651	EC:2.7.13.3	*resE*	OmpR family, sensor histidine kinase ResE
	K07775	...	*resD*	OmpR family, response regulator ResD
	K07652	EC:2.7.13.3	*vicK*	OmpR family, sensor histidine kinase VicK
	K07668	...	*vicR*	OmpR family, response regulator VicR
	K07778	EC:2.7.13.3	*desK*	NarL family, sensor histidine kinase DesK
	K03092	...	*rpoN*	RNA polymerase sigma-54 factor
	K03406	...	*mcp*	methyl-accepting chemotaxis protein
	K03407	EC: 2.7.13.3	*cheA*	two-component system, chemotaxis family, sensor kinase CheA
	K07693	...	*desR*	NarL family, response regulator DesR
	K10255	EC:1.14.19. 23/1.14.19. 45	*desA(FAD6)*	acyl-lipid omega-6 desaturase (Delta-12 desaturase)
	K03413	...	*cheY*	two-component system, chemotaxis family, chemotaxis protein CheY
	K03415	...	*cheV*	two-component system, chemotaxis family, chemotaxis protein CheV
	K00575	EC 2.1.1.80	*cheR*	chemotaxis protein methyltransferase CheR
	K11617	EC:2.7.13.3	*liaS*	NarL family, sensor histidine kinase LiaS
	K11618	...	*liaR*	NarL family, response regulator LiaR
	K04771	EC: 3.4.21.107	*htrA*	serine protease Do

K11619	...	*liaI*	lia operon protein LiaI
K11622	...	*liaF*	lia operon protein LiaF
K01034	EC:2.8.3.8/ 2.8.3.9	*atoD*	acetate CoA/acetoacetate CoA-transferase alpha subunit
K07636	EC: 2.7.13.3	*phoR*	two-component system, OmpR family, phosphate regulon sensor histidine kinase PhoR
K01035	EC:2.8.3.8/ 2.8.3.9	*atoA*	acetate CoA/acetoacetate CoA-transferase beta subunit
K11103	...	*dctA*	aerobic C4-dicarboxylate transport protein
K03367	EC:6.1.1.13	*dltA*	D-alanine--poly(phosphoribitol) ligase subunit 1
K03739	...	*dltB*	membrane protein involved in D-alanine export
K14188	EC:6.1.1.13	*dltC*	D-alanine--poly(phosphoribitol) ligase subunit 2
K03740	...	*dltD*	D-alanine transfer protein
K11633	EC:2.7.13.3	*yxdK*	OmpR family, sensor histidine kinase YxdK
K11634	...	*yxdJ*	OmpR family, response regulator YxdJ
K11635	..	*yxdL*	putative ABC transport system ATP-binding protein
K11636	...	*yxdM*	putative ABC transport system permease protein
K14983	...	*ciaR*	OmpR family, response regulator CiaR
K18345	EC:2.7.13.3	*vanSB(vanS)*	OmpR family, sensor histidine kinase VanS
K18344	...	*vanRB(vanR)*	OmpR family, response regulator VanR
K07260	EC:3.4.17.1 4	*vanY*	zinc D-Ala-D-Ala carboxypeptidase
K18940	EC:2.7.13.3	*arlS*	OmpR family, sensor histidine kinase ArlS
K07697	EC:2.7.13.3	*kinB*	sporulation sensor kinase B
K06347	...	*kapB*	kinase-associated protein B
K13532	EC:2.7.13.3	*kinD*	sporulation sensor kinase D
K13533	EC:2.7.13.3	*kinE*	sporulation kinase E
K02490	...	*spo0F*	stage 0 sporulation protein F (response regulator)
K06375	EC:2.7.-.-	*spo0B*	stage 0 sporulation protein B (sporulation initiation phosphotransferase)
K07699	...	*spo0A*	response regulator, stage 0 sporulation protein A
K11637	EC:2.7.13.3	*citS*	CitB family, sensor histidine kinase CitS
K11638	...	*citT*	CitB family, response regulator CitT
K11103	...	*dctA*	aerobic C4-dicarboxylate transport protein
K11614	EC:2.7.13.3	*yufL(malK)*	CitB family, sensor histidine kinase MalK
K11615	...	*malR*	CitB family, response regulator MalR
K00027	EC:1.1.1.38	*ME2(sfcA)*	malate dehydrogenase (oxaloacetate-decarboxylating)
K11616	...	*maeN*	malate:Na+ symporter
K07704	EC:2.7.13.3	*lytS*	LytTR family, sensor histidine kinase Lyt
K07705	...	*lytT(lytR)*	LytTR family, response regulator LytT
K07778	EC:2.7.13.3	*desK*	NarL family, sensor histidine kinase DesK
K11633	EC:2.7.13.3	*yxdK*	OmpR family, sensor histidine kinase YxdK
K05338	...	*lrgA*	holin-like protein
K05339	...	*lrgB*	holin-like protein LrgB
K09697	EC:7.2.2.4	*natA*	sodium transport system ATP-binding protein
K07706	EC:2.7.13.3	*agrC(blpH)*	LytTR family, sensor histidine kinase AgrC
K07717	EC:2.7.13.3	*glnK*	sensor histidine kinase GlnK
K07719	...	*glnL*	response regulator GlnL
K01425	EC:3.5.1.2	*glsA(GLS)*	glutaminase
K07720	...	*yesN*	response regulator YesN
K01104	EC:3.1.3.48	...	protein-tyrosine phosphatase
K01791	EC:5.1.3.14	*wecB*	UDP-N-acetylglucosamine 2-epimerase (non-hydrolysing)
K07707	...	*agrA(blpR)*	LytTR family, response regulator AgrA
K18350	EC:2.7.13.3	*vanSC(vanSE)*	OmpR family, sensor histidine kinase VanS

Table S19 Factors and control levels used for the design of experiments (DOE).

Parameters	Units	Symbol Code	Experimental Level	
			-1 (Low)	1 (High)
Petrochemical Effluent Dilution	cc	PED	1	10
Municipal Wastewater Amount	cc	MWA	20	50
Carbon Source	g	CS	0.5	5
Mineral Amount	g	MA	0.90527	3.62111

Table S20 Design of experiments based on orthogonal array L16

Test number	variables				Initial COD	First Day COD	Efficiency %	Second Day COD	Efficiency %
	PED	MWA	CS	MA					
**1**	1	1	1	1	6236.66±40.41	5226.66±56.86	16.19	4783.33±47.25	23.3
**2**	1	2	2	2	12860±121.65	10323.33±68.06	19.72	9866.66±230.94	23.27
**3**	1	3	3	3	22803.33±45.09	20218.33±70.76	11.33	17683.33±332.91	22.45
**4**	1	4	4	4	34913.33±132.63	33430±175.21	4.24	30343.33±160.1	13.08
**5**	2	3	2	1	15783.33±275.37	14153.33±215.71	10.32	10640±524.59	32.58
**6**	2	4	1	2	6753.33±100.16	6196.66±5.77	8.24	4326.66±97.12	35.93
**7**	2	1	4	3	50083.33±104.08	49140±121.65	1.88	47150±132.28	5.85
**8**	2	2	3	4	22100±173.2	20468.33±482.19	7.38	19271.66±998.2	12.79
**9**	3	4	3	1	23033.33±152.75	20483.33±301.38	11.07	19250±304.13	16.42
**10**	3	3	4	2	50133.33±251.66	48216.66±325.32	3.82	45900±173.2	8.44
**11**	3	2	1	3	7750±86.6	6623.33±390.68	14.53	4793.33±92.91	38.15
**12**	3	1	2	4	11186.66±162.89	10630±246.37	4.97	9826.66±310.05	12.15
**13**	4	2	4	1	51783.33±292.97	45016.66±828.15	13.06	33216.66±4479.49	35.85
**14**	4	1	3	2	49050±180.27	47166.66±152.75	3.83	45100±200	8.05
**15**	4	4	3	3	23916.66±104.08	22058.33±52.04	7.77	15383.33±5076	35.67
**16**	4	3	2	4	10863.33±118.46	10513.33±61.1	3.22	12880±5230.36	-18.56
**SP**	3	2	1	1	6560±170.88	5416±225.46	47.43	3833.33±162.58	41.55

Test Uncertainty= 0.058, Domestic wastewater COD = 846, Industrial effluent COD = 11300 (**PEA**: Petrochemical effluent amount; **MEA**: Municipal effluent amount; **CS**: Carbon source; **MA**: Mineral amount)
SP: Software prediction
